# Supplementary material for: Dietary adherence and program attrition during a severely energy-restricted diet among people with complex class III obesity: A qualitative exploration
Source: PLoS One. 2021 Jun 17;16(6):e0253127. doi: 10.1371/journal.pone.0253127 (PMC8211265; doi:10.1371/journal.pone.0253127)
Supplement: S1 File — (PDF) [file pone.0253127.s001.pdf]

<u>Semi-structured focus group and telephone interview questions</u>

We appreciate that you have taken the time to talk to us today. Before we start recording I would just like to remind you that today's interview will be audio recording, is that ok?

If yes start recording and start questions. If no, find out if its ok to take written notes, remind them how this information will be used. Ask if they are ok with their quotes being used in research publications. Terminate the interview if a participant is opposed to the above.

**Question**	**Purpose**
Are you doing anything for weight management at the moment?<br>➢ What are you doing?<br>➢ If not, why?	Provides context
When you were initially told that this service included the use of meal replacements, how did you feel about starting a meal replacement program?	Past experience barriers, emotional response
Thinking back at when you did the diet program, could you describe what this experience was like for you?<br>➢ Did your experience match any expectations that you had of the program?	Beliefs about how they saw the programme, themselves and expectations, judgement on adherence
Did you have any difficult days where it was hard to follow?<br>➢ What about a bad day look like?<br>➢ How did you cope on these days?	Program compliance
Did you add extra protein?<br>➢ How are you taking it?<br>➢ How you feel about having the extra protein?	Provides context
How long were you on the meal replacement program and what type of results did you get?<br>➢ How satisfied where you with these results?	Provides context

What did you think affected your ability to stick to the diet program either positively or negatively?<br>➢ If you did feel like you weren't able to stick to the diet or finished earlier than you intended, what do you think got in the way?<br>➢ What are the factors that you thought helped you stick to the diet?	Past experience, exploring social, cultural barriers, diet structure, taste texture of products, diet enjoyment
Do you consider achieving results, such as weight loss an important factor in motivating you to change your dietary behaviours?<br>➢ Can you think of any other motivating factors that are also important to you?	Exploring drivers
How was your social life affected by the program?	Exploring social/cultural barriers
What personal skills do you think are important to be able to stick to the diet plan?<br>➢ What helped you to develop and implement these skills?	Self-efficacy
At any time during the program, did the way you feel about yourself (good or bad) impact on your ability to stick to the meal replacement plan?	Self-efficacy
Did you ever feel like dropping out of the program, why? and why did you stay?	Self-efficacy
What do you think about the cost of the VLED program?<br>➢ How did this compare to your weekly grocery bill?	Affordability, socioeconomic factors affective adherence.
In regards to how the VLCD group sessions were delivered, how did you feel about the group sessions you attended?<br>➢ What parts helpful or unhelpful?	Evaluating program delivery and points of contact
Were you satisfied with the frequency of appointments and support provided at the service?	Evaluating program delivery and points of contact
Is there any feedback you'd like to give or share with us?	Exploring service gaps

That concludes our interview today, I really appreciate your input. I have now stopped audio recording. Just a reminder that if you change your mind about participation or the use of de-identified quotes being used for research, please let me know so I can delete the content. Thank you and have a lovely day
