# Supplementary material for: Dietary adherence and program attrition during a severely energy-restricted diet among people with complex class III obesity: A qualitative exploration
Source: PLoS One. 2021 Jun 17;16(6):e0253127. doi: 10.1371/journal.pone.0253127 (PMC8211265; doi:10.1371/journal.pone.0253127)
Supplement: S2 File — (PDF) [file pone.0253127.s002.pdf]

Participant transcripts

Date: 15.12.17
Interview time: 71mins
Focus group -two participants ID: 1 & 18

I- How long did you use the meal replacements for and what type of results did you get?

R1- used for 12 weeks, lost 14.5kg. Have scales at work and I am down 15kg as of a few days ago I weighed in at work.

I-Did you try to lose weight before starting this program?

R2- I started the program 2 years ago, and had 7months off I was in NZ for 3 months. When I got back it was close to Christmas and it was about 6.5months before I got back into it and on to it again. Seeing the dietitians, I initially lost 10kg then I guess I put that back on again. It wasn't until I started the 12week Optifast program that I lost a significant amount of weight again.

I-Are you still doing the Optifast program and meal replacements?

R2- Yes so now I am doing 2 meal replacements and 1 meal, that's no carb as in no bread, rice etc.

I-Did you find doing the Optifast program help you figure out that what that last meal was?

R2-Yes, significantly. It was a conscious decision not to introduce carbs again. Sometimes you do need to have that little bit extra. But I have found that I don't miss it much because I have found more volume and interest in eating more vegetables. It's been interesting trying to come up with ways to cook vegetables, so that it's not plain and bland boring. I have tended to use my oil allowance to roast vegetables or stir fry them with no meat. Used various herbs, spices and garlic to create variety in the vegetables.

I-How long have you been on the two meal replacements plus a meal?

R2- Only for the last few weeks since I've seen XXXX. It will be interesting to see how I go for this maintenance phase. Then I think I have to do the full Optifast again just before surgery. Which will be it will be easier to do just before surgery because I've already done it.

R1- I've also been on the program for 12 weeks and lost 45kg. I've been on 5 meal replacements on the intensive phase and the vegetables. I've been pigging out on the veggies and it sustains me. I'm so impressed with the results. I'm trying to do everything to my best so I can get towards my goal. I'm surprised how effective the program has been. I was at the obesity program at XXXX hospital for 12months and didn't lose a thing. The minute I came here I lost 9kg in the first 2 weeks. I was ecstatic. But I won't go and buy a burger right now because of the results I have achieved.

I- How did you both end up coming to the service?

Participant transcripts

R1- By accident my physio wanted me to access hydrotherapy, the only way I could come to hydro was to be involved in a treatment plan within the hospital. That's how I ended up at MOS. I initially got involved with Dr XXXXX program at XXXX because I was too heavy. I was over 200kg. At the moment, I am 220kg. I had just dug a hole so deep to get out of my hole looked insane, if an obesity clinic can't help you, you know you're in trouble.

R1-That's how I am here. I find general practitioners or people in the medical industry, just aren't aware of these kinds of services. I'd go to the GP and I wouldn't get a referral. Now I tell people if you have any friends or family with an obesity problem give them my number so I will tell them what they need to say to their GP.

R1- I was at XXXX hospital and all they tried to do was keep me on a low calorie diet, and as you know low calorie and this is completely different.

R2- My experience was completely the opposite to you. I was GP referred, my GP new about it. He filled the forms in and got me to sign it and he faxed them across. He got me to sign a commitment thing as well and I got contacted by MOS. And the journey began.

I- How did you feel about starting a meal replacement program?

R1 - When I first started it was purely diet only with the dietitians. To be honest I did get very disheartened because I completely changed my diet, was eating less and different types of food and not eating a lot of weight. I do have to say though I love ice cream and I do love some chips and stuff like that. I wouldn't say every night, and that was my downfall, and when I cut that out and I still wasn't losing weight that was very disheartening. When I started the VLCD and I lost 5kg in the first 2 weeks, then I thought this seems to work.

R2- It was interesting to read the product packets to see the difference between the LCD or the VLCD to make sure I was buying the right one. I didn't feel particularly hungry on it. I would have a shake for breakfast, a bar, I loved the chocolate fudge bar and a soup at lunch, I may have had time for another bar, then soup again for dinner. That's how I did it for the 12 weeks. That's how I did it.

R2- I think you get into a routine or structure. Once you get into the structure or routine, you say to yourself – well I liked that, I'm going to cook that one again. I'm usually a recipe person, if I have a recipe in front of me I just have to cook it. It takes the thinking away. When you're a shift worker it makes life so much easier. All the work is done for you all you have to do is cook it, which I find is so much easier about thinking what I have to have.

R1- I was scared as hell butterflies and absolutely nervous because I had a big job ahead of me. I had tried so many diets and had failed. When I had come here for my first interview, I was in a mad rush that I didn't have time for breakfast, and I was starving. XXXX she mixed up a shake and I sampled it, when I had the shake I recognised its potential straight away. I thought wow that's killed my hunger, this opened my mind, I thought this has potential. I then came in after 2 weeks and dropped 9kg and I was in tears. I saw how empowering it was, and that is what drove me. Once you can get people to try a sample they can at least try it for 2 weeks. That's the impact it has on me. I am now so determined that nothing will get in the way.

Participant transcripts

R1- I've even been at Christmas parties, where people have been throwing plates of food around at me and I've been sitting there with nothing twiddling my thumbs and they see me. They ask me if I want food and I say no. Then I ask them if they have diet drinks, and they typically don't so then I ask for a black coffee and take out one of the bars.

I- They sound like a bit of a saviour?

R1- Well, yes, they are because you go along to stuff and there's nothing there you can eat. You go to a friend's place and they bring out cake or whatever because they feel like they would be offering you something. You can just say, no thankyou I have my bar.

R2- You take it along with you and then they don't feel embarrassed because they can't offer you anything because they know that you are on this journey. And I have to say the support from my friends have been fantastic.

R1- The other thing is if you go out to eat, I thought there's nothing here I can have. But then I broke it down and thought, well, I can have that. That was a couple of field mushrooms, salad, poached eggs, and smoked salmon on sour dough bread. So, what I did is I left out the bread. It's just about digesting and deconstructing the meals that are on offer and determining what you can have.

R1- Being out and busy places some strain on the diet it makes it difficult. I travel with a bar, but on the odd occasion, by accident I stayed at family's house overnight. But I went around to subway and found they had a large salad with chicken fillet without dressing. So, subway is useful for people in an emergency situation. Then I went to a service station and found a protein bar, which had a similar composition to a meal replacement bar and thought that would do. This helped me make it through. I don't think I broke my diet that day, because I had protein choices.

I- Do you find it socially isolating being on the meal replacement program?

R1- I think it really depends on what type of friends you have and how supportive they are. You go to some of the salad bars, like sumo salad and you look at the calorie and carb count and you look at them and think, well, I can't actually eat any of that. There is one place that does kebabs and stuff that make salad bowls, and I get the vegetarian salad with tofu. That's a nice decent size, and you get enough vegetables. There are options out there, but not many options and I guess that's the hard thing. The easiest thing is to deconstruct the menu.

R1- My brother tried to help me out when I stayed at his place, and he made me a salad. It came out with olives, corn and dressing. I said, I can't eat olives, he told me to just pick them out. But I said no, I couldn't, so I basically rejected a salad would you believe? Just shows you how committed I am to this and I wasn't going to put my diet at risk.

I- What do you think is your overall experience is of the very low energy diet?
R1- AAA+

I- So it was positive?

R1- Absolutely

Participant transcripts

R1- Look when you are on it and seeing the results that's motivating, but when you don't see results you then become disheartened then you mind kicks in and you think, well why the hell am I bothering?

R1- Results are very important

R2- So when you are on the VLCD and you start seeing the results within 2 weeks. You go, ok.
I think the trick is don't weigh yourself every day. Only weigh yourself once and week or once a fortnight. That's why I don't have scales at home. Normally I would weigh in every day and not lose anything and then think, why bother? So now I do it at work on a Friday. I get on ok, I've lost, ok, now I haven't. But then you work out over the entire month, and you work out your results according to what is typically seen on this program 0.5-1.5kg for females or up to 2.5kg for men. Then you look at your results and think, well ok, it is working it is coming off.

> **I- Do you think your opinion of the VLCD would change if your end goal was not surgery? If you thought this was just the program in itself?**

R2- Probably not. I was put on the program not knowing that I was being built up for surgery. But when I started the VLCD the first few weeks, month, surgery was not discussed. Speaking to other people in the group I knew that starting the VLCD was done prior to surgery. It wasn't until a few weeks past that surgery was discussed and I was told about surgery. Then I was told your name was put forward at the surgery meeting and I was told my surgery appointment was XXXX. And then there was the building up for that, but then you have that gap from when you finish the VLCD diet, then you have the gap between finishing the diet and surgery. I also think this is a hard time to do the diet because of Christmas coming up you've got the festive season coming up.

R2- You think well, then what can I have. I don't drink alcohol so that's ok. I was saying to XXXX the other day, I'm not going to deny myself pudding with custard.

R1- Well I do

R2- And that the idea of it. Every one's an individual and I know that having that I won't want that on any other day. But on that day celebrating I'm not going to deny myself at that celebration with friends. But what I am going to feel challenging is giving up carbonated beverages after surgery because I don't like the taste of plain water, like tap water. That's why I carbonate it. I've gone from drinking soft drink to drinking the light diet right cordial. I will drink the water with a little bit of sparkling in it. It will be my biggest challenge to give that up post-surgery. You stick to it as much as possible. But again, it's everyone's individual mental capability that gets them through it.

R1- It's what I've been through in life that makes me so determined. I was at the point in my life where I was almost completely incapacitated. I used to go out walking and always have to look for the next possible seat. I've had it up to here, I am not going to let anything compromise this. I've been in so many family conflicts that you just want to comfort eat afterwards and I fight with myself. Damn it you don't want eat anything! There are periods in my life where I have faced the near impossible, where I have just said BUGGER IT! I tend to

find that I'm sometimes always successful in life because I have always carried on regardless. There comes a point with me where I switch where I am 100%.

R1- You know when I go to Christmas parties, yes, I would like to have a little nibble, I look after my elderly mother and I prepare her meals. I even buy prawns for her, but I won't even touch one prawn, and prawns aren't harmful on this plan. But I refuse to eat anything that is not on this plan. You are dealing with a person that's highly driven, and so now I feel like I have been given the tools and I am basically very determined.

R1- On the topic of bariatric surgery. I don't want bariatric surgery. I have my reasons. When I was at XXXX hospital the Dr was trying to convince me to have surgery. At 260kg I'm sure I was a good candidate because I am in the top scale. But I know what I was doing wrong in the past eating Mac Donald's and I have lost weight in the past and regained it back several times. It was similar to giving up smoking.

R1- But where I found MOS to be useful was in their lectures and education with the program. They tell you what foods you can and cannot eat. I now know that I can pig out on vegetables, but choose the non-starchy vegetables such as broccoli, cabbage and eggplant. For me it's about knowing what I can eat, which I didn't know before.

R1- I'm at the point now at 220kg, my life has really changed because I am now doing things like, being able to wipe my own backside. This was like wow. I couldn't do that before. Imagine at work, my work colleagues where complaining about my smell, and the reason was, was because I couldn't wipe my own backside. So, I would go into the disabled toilets and lay down on the ground because when you are obese the only access is to lie down. The other day I was in the shower and I thought wow, I can actually wipe my bottom. Normally I wouldn't normally be able to do this. I would have to get out of the shower and lay down on the bed and use wet ones to clean myself.

R1- I can't even go to the toilet like a normal man, like to a urinal. I've had it up to here with it, I'm sick of it. Enough is enough.

I- Sounds like this diet came around at just the right time?

R1- My life is very complicated. Because of my obesity I lost my job, I couldn't get back on to my feet. I ended up becoming bankrupt, lost my house and couldn't get back to work. If you look at my resume you would see I am a smart guy. By life has basically become destroyed over this. I am somehow lucky to recover. Now that I can see this opportunity, so I have latched on. I think it's the same as when someone comes from a poor childhood, they go into business as adults and make lots of money, and they know how it feels like to go hungry. My lessons in life, I have applied for 600 jobs, in the past I've had a very senior role in some very prestigious organisations. But what would happen is people would just take one look at me and think, this guy is just a time bomb waiting to go off. They wouldn't take the risk of employing me. After 600 job applications, I thought, well I am probably not going to get a job. For me it's been tough, because I know what I have been capable in the past, and I just ended up becoming an unemployable pile of shit. Obesity is a serious problem.

I- How do you feel now having lost some of the weight?

Participant transcripts

R1- Very empowered. I have big dreams and ambitions, I am trying to invent myself as a documentary film maker. When I was 160kg and I was as fit as the best of them. I used to go on search and recuse exercises with the XXXX police force as a packet a day smoker.

R1- My Dr used to shake his head at me, I didn't have diabetes or high blood pressure and I smoked. He would make me have my tests done and be amazed that I didn't have any problems. I know I feel better just losing a little bit of weight, at the moment I feel like anything is possible. I plan to go into film making now that I have lost some weight and have big aspirations in that area. This goal has given me something to aim for, and has given me the sanity. If I didn't have this I'd probably look for something to shoot up with.

R1- I am now on a disability pension and I live in XXXX and I see how people living around me freely admit they drink 3 bottles of wine every night and smoke cigarettes. They seek food from charity organisations. That's another problem I have I can no longer access food from charities because they don't have the right foods for this plan.

I- What do you think contributed to your ability to be able to stick to the program?

R2- I think it was the continuity of the program, what I had to cook and what I had to take to work, and simplicity of the diet. At that stage you aren't going to the shops and walking past the biscuit isle and saying "oh I just want that" and put that in and that in. You go to the chemist warehouse and you've already bought it, so those temptations aren't there. The hardest thing I had to do was choose which vegetables I was going to have and the access to the diet.

I- What do you mean by access, as in having the product with you at work made it easy to get to, or do you mean access, as in where you purchased the product?

R2- Well I mean, both. I am a big chemist warehouse fan and they usually have, you watch and you see different chemist warehouses have favours and what they stock and what they actually have on the shelf. But yes, it's just the simplicity of going and getting your shopping once a fortnight at the chemist. Well, I have to go and get my medications anyway, so I'm already there and that was it. Then it was a quick trip to the shops to get my vegetables.

I- And what about the affordability side of things?

R2- I guess the affordability, I mean I work but in saying that I live on my own and pay rent, so there are bills to pay. By the end of the pay period there isn't a lot left over. I found that Optislim was cheaper than the Optifast. But with the Optislim you had to watch what was the VLCD and LCD and what to take and differentiate between the flavours of the soups and find out which ones I liked.

I- What about the comparability of how you used to eat, with the affordability on the program?

R2- I guess it's cheaper because you don't have to buy your meat or milk. I've gone from drinking coffee with milk to long blacks to tea with a slice of lemon. The affordability is that it works out to be cheaper than doing your weekly shop.

Participant transcripts

I- What there any challenges that got in the way to your ability being able to do the program? Past beliefs or anything like that?

R2- Well, no because you can't eat anything on this plan.

I- So it's almost as if it removes the choice?

R1- Well yes, it does. And if you don't have it in the house you don't eat it.

I- How about you XXXX? Did anything affect your ability in being able to do the program?

R1- I am very lucky in that I have a very loving and supportive mum, who said I will support you son. So, I order mine from Brightsky which is supposedly cheaper. My mother supports me to do this diet, she pays for it and I help her with other things such as cooking for her, running errands and being her personal sectary.

R2- I was in chemist warehouse the other day too and the Optifast was $60 for a packet of 18, it was $48 for a packet of 12 bars, I said oh my god! I don't know because I buy them in bulk from bright sky. What motivates me is the school of hard knocks, I used to bush walk and I used to love being outdoors. I did a bush walk at 268kg, a 200km track. No one thought I would make it and I realised I may have bitten off more than I could chew. But then I got to the top of the mountain and I was in survival mode. When I got the visitors centre I got a standing ovation because no one thought a man of my size would make it. I seem to be pretty strong.

R1- I've been to Christmas parties, and as I leave people give you goodies like chocolate, biscuits, chips to take home. Now I take them home and I don't touch them, because you know that's just me. People who know me, know I go crazy when they see me in this mode.

I- Do you think it's fair to say, one of the things that helped you stick to plan is being very rigid?

R1- Yes, extremely because I already decided that I had lost the weight. It's not a matter of if, it's a matter of when. I found the diet very practical because it's in the satchels. If it was a diet where I had to eat this, then eat that and having to manage that it would be too hard. I find that having the teaspoon of oil rather silly, I eat my fats via eating nuts. I am supposed to have 7g of nuts, but because I am a big fellow I am going to have 12g of nuts. I just decided that's what I was going to do. I measure them out via the use of food scales. I have pumpkin seeds, sesame seeds.

I- Do you feel having the variety of nuts have helped you?

R1- Yes, I do feel that's important. I approach it as a treat. I get more satisfaction from eating the nuts rather than oil. I find the nuts as a reward every day.

R1- Mum is very good tries to not eat in front of me not to tempt me. She hides in the kitchen and I don't realise what she is doing but she eats in secrete to help me. But you know I love

watching cooking programs and researching different recipes on the internet. I do it all the time, I am able to detach myself, even though I love cooking. I am interested in food, and buy food to cook for my mum. But I don't eat it, with me I have the determination to do the diet. A point has come in my life that nothing is going to stop me. It's not a matter of if, but a matter of when.

I- Do you think the way you feel about yourself has affected your ability to stick to the program?

R2- No, the reason why I did the program is I suffer from a number of medical conditions XXX in my spine, and I've already had injections for this and carrying the weight I just can't sustain it.

I- What do you mean, is it because you are in pain?

R2- Yes, and you can't live off anti-inflammatory medications all your life, because that's not good for you either. I also have XXXX so there are certain foods I can't eat. When I was first XXXX, then at another point I had become septic. I tend to stay away from food sources that may flare the XXXX such as nuts and seeds. Because of my medical conditions I really have to watch what I eat. Along with doing this.

I- So there are two sets of restrictions going on?

R2- Yes, there are two sets of dietary restrictions.

I- Did you find that challenging to navigate through?

R2- It is, but it saying that I get cucumber and tomato and de-seed them. So it's like I've got two food challenges, I wouldn't say "diet". Just food challenges. Yes, I can have that or no I can't have that because of the seed content, and you just avoid them because they get caught in the food pockets. In saying that, being Dx with XXX disease was actually a savour because I was Dx with an XXXX at the same time. I've had that challenge too.

I- Did you say the health scare motivated you?

R2- I was diagnosed with XXXX, and it was benign, so I was lucky but you don't know that at the time. The other challenge was it put me into early menopause, because I had a XXXX. You don't have the hormones either. So, then there was that challenge too, to go on top of it.

R2- And as I said I work a rotating roster night shift/ day shift and whatever. And some of the girls, I've told people at work that this is what I am doing. And I have nothing to hide. I know some people who are on a Facebook page for gastric bypass and sleeves, can I say though that I'm a troll. I just scroll through everything. And because of my XXXX background some things you see you think are you for real, and others I think, well that's quite interesting. And you know what you realise there are so many people out there on the same journey as you. And I found this actually quite good.

R2- I've recently had friends who have recently been sleeved. And one of them is going through a bit of a rough time at the moment, she's had an abscise and I've been sorting

helping her giving her ideas on what to eat, giving her ideas on how to prepare her vegetables such as increasing the iron content by making up a gravy using meat juice or adding an egg to her veggies.

> **I- Do you think having skills like, knowing what foods are made of, is an important skill to have on this diet?**

R2- I think it is.

R1- Yes so do I. In fact, I think with me will transform the way I eat. With me what I was big on was the organic health food and I'd pile on the organic honey and eat what was "health food", but I was eating cashews and almonds by the bucket load. But now what I understand is even healthy foods are dangerous, like honey for example. And these foods need to be eaten in moderation. And these foods, what I would consider "pig out foods" pigging out on broccoli and cauliflower, and I actually find those foods satisfying. But what I used to do was boil potatoes and not peel them, and eat half a pot of potatoes and pour olive oil over the top and be satisfied that it was a healthy meal. Because its olive oil. The lectures have been very useful. The stuff that you did on the glycaemic index I found was very useful, I took it all to heart. I purchased the Doongara rice and I bought the stevia and low GI sugar for when I come off the diet.

R1- I've taken all the lessons and made notes in my note book and research it, to go a little bit further. Because on the VLED that I'm on there's a prescribed foods list that these are the foods I can eat. Whilst it hasn't been said, I assumed that's the foods that I will be eating later on when I come off the diet.

R1- What I did was I purchased fish poachers, so I bought a small one, so there is the intention of how I will be preparing and eating my food in the future. Steaming food, I enjoy steaming food.

R1- Parts of your lectures have really stuck with me for example what your plate should look like and what your meat should be, for example ¼ meat, ¼ starchy veggies and the rest veggies. Now I wasn't aware of that 6months ago.

R1- I understand it's common on VLED that when you have rapid weight loss, people can very rapidly put the weight back on. So, for me it's really important that once I get the weight down I keep it down. Because I've been there before, I've gotten the weight down when I was in my 20's. In my 20's I was a 120kg and I thought I was a pig of a man and I lost 30kg and I was very fit, I used to do long distance running. It's like being a former smoker, and I used to be a former smoker you need to understand the phases. Sometimes I find myself smoking again for about a couple of weeks and I say to myself I am going to give it up again. So I fully understand the withdrawal thing.

R1- I tried the juicing diet, but found there was no protein. So I couldn't sustain it. My attitude to food has changed significantly due to the lectures. You people give me these print outs and I go home and study them, for example it tells me I'm allow 5g nuts per day or 1 carrot and yet I'm pigging out.

> **I- You feel like you're pigging out?**

Participant transcripts

R1- Well yeah, I get to fill a whole pot with cauliflower, broccoli you know what I mean, onions. You say a cup a day, and I eat a pot a day. But look at me, I've lost so much weight.

I- You say that the lectures helped you, so I see that as a supportive thing in the service. What other things would you like to see in the service that would support you better?

R1- The group VLED sessions are very useful because you get to see where everyone is at. I got to see where mentally I was at compared to different people. Because I am extremely determined, so when I heard people say they caved into food in different situations, that was a surprise to me, I thought wow I am in a completely different world, because nothing will break me.

I- So the interaction with other people and maybe the comparison helped you?

R1- Yes, and I thought what might be useful is if you produced an instruction video for people who had trouble accessing it and people who have never heard of the VLED. I was surprised to hear about it, and I consider myself an informed person. I've heard of all the diet Atkins, Pritikin diet and I knew several of the other diets and this was completely new to me.

R1- I was discussing this with Dr XXXX. I wasn't accepted into his program because of my weight, now his program is a little bit different in the sense it had a physical component like a boot camp of such. And I know that I need to get physical, not that I'm ungrateful of what MOS has done, but unfortunately MOS doesn't have a gymnasium that I can turn up to and use.

I- What did you find in the service was supportive or that you thought there was a gap?

R2- Exercise, is my thing. Because I work and when I get home I'm really tired the last thing I want to do it exercise or go for a walk. But if I'm meeting someone, and I often meet a friend at the beach to use one of the ocean pools, my thing is swimming, I'm not a gym person. I can't stand gyms because I find everyone pretentious in there and I've had nasty experiences where people judge you. And as much as they say, there not judgemental its bullshit, they are and they do. Maybe an exercise program where people can join in, like we come along to the diet groups, we can come along to as part of the group would be a great part of the program. So that you could meet and form a group and go for a walk or a swim. Something like that. The same as you would see a dietitian.

R1- The advantage I see of the program at MOS is its handy for working people because it doesn't impact on your time. Whereas Dr XXXX program requires 3 visits a week so for someone like me who is on a disability program it's great because 1. It's a distraction, you eat because you're bored and you have nothing to do. 2. it's the interaction with people. Different horses for different courses. That program may not be suitable for everyone if you're working.

R2- This program here at MOS doesn't have an exercise program that is available to people and I would suggest, that it would be useful for some people. But I understand that comes down to funding, it all comes down to the dollar.

Participant transcripts

R1- Not only funding, but even with me, it becomes and OH&S issue.

R2- But even if it's not a gym. We could all meet, I mean there is an oval just out there and we can just walk around the oval or I don't know if you have access to a pool we could use.

I- We do refer people into a hydrotherapy program

R- Yes but referring them into and getting into it is hard

I- Yes, it is very difficult to get in, it is a bit hopeless

I- Is there anything else you would like to tell us that you liked about the program?

R1- You can pass on my gratitude to everyone at MOS. I have personally wanted to thank Prof XXXX, who probably came up with the program, and the people here because without you I'd be in a much worse place. You have been able to transform my live and I will be eternally grateful.

R2- I think it's a shame that other area health services don't offer a similar service as the service here. Even at the hospital I work at nothing is offered publicly, it's all private. There are so many people out there that need to be on a program and need help.

I- Well, things are changing, there are new services opening around NSW and further out west so hopefully it will change.

R1- Maybe there is something you can do something online, like an online learning platform. This would help people out of area access the information easily.

R2- Accessibility is the issue, this is one of the few Medicare funded programs. And as I said two girl friends of mine have been sleeved at private hospital, and that's privately funded by them. So, something that's accessible financially should be more of them, so it's easier for people to access them. You have all these area health services, why don't different area health services have a similar program running?

I- Well, it is changing. But it has taken a long time.

R2- Well that would help the adherence and the drop out. Because the accessibility is difficult.

R1- I'm lucky I live 10min from the hospital and that's why I've attended regularly and that's helped. But today I came from my mum's house in Casula and I was rushing and almost late.

I- Just on the adherence part did you find frequency of coming to the groups helped at all?

R1- It's like going to church, people can be religious but by going to church every Sunday they reinforce their beliefs. So, kind of doing the pilgrimage.

Participant transcripts

R2- I found for me doing shift work and work full time, coming monthly was good. I would use my sick leave to attend the groups. I thought I have the time accrue and this is what the sick leave is for. Just do that. I think for me personally was enough of a distance apart to see the difference. You do your VLED for a month, stick to it, come in see you dietitian, you weigh, oh great you've lost weight- good now keep it up. You still known to everybody, even though it's been 2 years, it just keeps it real. It keeps it going.

I- Are either of you adding any extra protein to your plan?

R1- Yes, I do. I got on to protein perfection, I don't like the milky protein. This one you mix it with water and it makes a cordial type thing. It's called protein perfection, its 15g of protein per scoop.

I- Do you find adding that in helps? Have you ever had the shakes without it? Have you ever had the VLED without protein?

R- I've always had the extra protein on the side, not in the shake.

R- I try to get it out of the whey. I don't always want to have the shakes so what I do is, in the morning, and I've been assigned to having 9 scoops a day, so in the morning I'll have the 9 scoops in my shake and that gives me more flexibility in the day. Because it's very difficult to have the protein with the bars or the soup. Initially what I did was 3 shakes per day, but what I found was that this didn't make me happy, so I tried to cram my protein into 2 shakes and this then gives me 3 meals I can play around with.

I- Do you find that your hunger is more sustained when you have that extra protein with it or near it?

R- I don't think the extra protein made much difference

R- I can't honestly say, because intellectually I know that protein is meant to satisfy hunger. So to answer that question I don't know if I am answering that academically or personally. I can't give you an honest answer.

---Interview end---

Date 5.2.18
In-person interview ID: 2
Duration 50min

I-Are you doing anything for weight management at the moment?

R- I am back on the shakes. So, I have one in the morning and at dinner time I have a little piece of fish or chicken the size of my palm and veggies. The right veggies, and then I have a piece of fruit like an apple or a pear. I used to have strawberries.

I- How long have you been back on it for?

Participant transcripts

R- I've been back on it for 1.5 weeks, back to normal. Before that I had a few things happen in my life.

I- That's ok we know that sometimes life happens. So, let's rewind and take it back to when you first started. How did you end up coming here, how did you find out about this place?

R- I went to my doctor who recommended that I go see you guys. The she wrote a referral and I called up and booked and appointment. It has been a good success for me, from all the other diets I've done, this one tops the cake.

I- What other diets have you done in the past?

R- I've tried Weight Watchers, Lite N Easy, shakes, pills... Everything almost. I found with this diet I lose more and I feel better, especially at the start. It doesn't make me feel sick like the other diets do.

I- How long did you do the intensive phase of the diet for?

R- I started as soon as I booked the appointment, I went to the shops and bought the shakes. I tried the Optislim and thought it wasn't bad and then tried Opifast and thought, that's not bad, but Optifast is a bit expensive. I have heard that both brands are basically the same, except Optislim you can get two boxes for $50 this lasts 2 weeks, whereas Optifast is $60 per box, which lasts less than a week.

I- We ask people to do the diet for 12 weeks, how long did you do it for?

R- I did it for longer than 12 weeks, I was happy to continue past the 12 weeks. Then I came in for a consult and I was asked if I wanted to do a meal, and I said yes. I think I was ready for it then.

I- Then you did the partial meal replacement after that, how long did you do the partial meal replacement for?

R- I did that for a while, then in the last consult, then I did the 2 meals a light lunch and light dinner. But then just recently I've decided to go back to 2 shakes and 1 meal. I find that I lose the most about of weight with 2 shakes and the light meal at dinner. Then I know ok, I know what to have for dinner, I'm still not worried about two meals. That way I can learn how to control my meals.

I- **Do you feel having to only concentrate on one meal easier?**

R- Yes, even though I have 2 shakes I know exactly what I can have for dinner. I don't have to worry about breakfast and all the rest.

I- When you were taking the 3 shakes, did you take any extra protein?

R- I took the protein in the morning

Participant transcripts

I- Mixing it into your shake?

R- Yes, I mixed it in my morning shake, I didn't put it into lunch. I found that the protein tasted a little bit funny.

I- What protein did you use?

R- I used Beneprotein, I put in 2 scoops and then 1.

I- So, then you only did the morning 2 scoops and didn't do the one later?

R- Yes, I was finding I was getting reflux with it. I have lactose intolerance, so any more than that was making me feel sick.

I- Were you using the protein for the entire intensive phase?

R- I was using it for a while, because I had to purchase so many tins in bulk. So, I stuck with it for a while, my sister and I bought it together, I said to her do you want the remaining of the protein and she said nope! So, I asked a friend if she wanted it, so I gave it to her. She found it was right for her.

I- Since you've started back on plan did you resume taking the protein?

R- No, just the shakes

I- Did you feel a difference in hunger when you had the protein, compared to when you did it without?

R- No, not really. Before the protein was helping, but now because I'm used to it, my brain is saying that's it. That's all you get. Now when I'm hungry I just have water. So now I think, just don't do that because you'll just put on. I think that's what my brains telling me to do, and I don't want to do that anymore.

I- Is there any other "extras" you are adding to your diet plan during the intensive phase?

R- No, what XXXX told me to have, is it. I had vegetables, like 1 carrot per day. And I had lettuce, tomato and lettuce is nothing, and I was having a bowl. And I was having strawberries, because that was the only fruit I was allowed to.

I- Sometimes people have like chewing gum, broth or diet cordial. Did you use any products like that?

R- No, just water.

I- How much weight did you lose all up?

R- I think it was about 20kg.

I- Is that the most you have ever lost doing a diet plan?

Participant transcripts

R- Yes. I started at 150kg something, and now I am 130kg… that's a big difference

I- What was your overall thoughts of the diet?

R- I do I like it a lot, because it's the only program that's helping me. All the others were not convenient for me.

I- Why do you think that is?

R- Well, I do I actually feel better. When I was in the 150s I couldn't do nothing. I was feeling tired, not motivated, I was down. Doing this had built my confidence up, I've started walking again, even though I have a bad back and bad knees. You know it's from doing nothing, to doing stuff now is completely different. My kids and my family have seen how much I've lost and they are proud of me and I'm proud of myself. It's just been the last couple of weeks that have been a bit bad. It's been a bad situation with my dad [cancer diagnosis], but other than that I've been ok.

I- Yeah sometimes life is challenging. What other barriers did you experience during this diet?

R- The only barrier, is that I wanted to do more and I couldn't, like exercise. I had back and knee issues that prevented me from doing that, even though I wanted to. I know that I could have lost more and instead of 20kg, I could of lost 40kg. When I was younger I was always 2 digits, and now I'm 3 digits. And that's one of the bad things, I want to exercise, but I can't. I'm now starting hydrotherapy, and I find that's pretty good. I make sure I do my two classes a week. It loosens my legs and I don't feel it, which is good. The hydro instructor said I could start trying the aqua aerobics classes, without the weights, so I am going to try that soon.

I- What did you use for motivation to stick to the diet?

R- I hopped on the scales every couple of days and kept track of it. It was a very good motivation.

I- At any time did you think the way you felt about yourself, like feeling down, did that affect your ability to stick to plan?

R- Sometimes yes, and no. If my brain says don't do it, then I won't see the big picture in the long term. I want to see the picture I'm the long run, I want to see the person that I used to be and I am going to keep myself motivated to get there because if I stop doing it I'm not going to get there, and I want to see myself get there in the long run.

I- You said sometimes it did, so give me an example of what stopped you from doing it?

R- My knees, they were hurting too much. I wanted to walk and I couldn't. My kids wanted me to go for a walk with them and I couldn't. My knees are so sore I can't go.

Participant transcripts

I- You said recently that you fell off the wagon, because your father was so unwell. Was that the only time and how long did that last for?

R- It latest for 4 weeks

I- And what did that mean, did this stop you from using the shakes?

R- No shakes and no exercise.

I- And junk food?

R- No, no junk food. I'm not a junk food person. I just ate more fruits, like things that you're not supposed to have.

I- Like more carbs?

R- Yes, that's what made me put on.

I- And when you went back on it, was it hard to start again?

R- No. Having hardly anything and just a shake is easy, just staying off the bad stuff like apples and sugary stuff.

I- What bought you to the point where you were able to get back on it again?

R- Putting on my clothing again. The reminder that my clothes didn't fit. Looking at the dress that I wanted to put on and not being able to fit in it, it was tight. It made me think wow, you need to get back on it. I got a dress and its size 12 and I want to get back into it.

I- You said that thinking about your dad made you feel sad and led you to fall off the wagon, what strategies do you use to get back on the diet and stay on it?

R- I think of myself, not do what I did before. Yeah, my dad's sick, but don't wreck yourself or your life, because then it will wreck the kid's life. This then motivates me to keep going.

I- That's a good motivator to use. Do you think the weight loss has motivated you to keep going? For example, if you didn't lose weight would you keep going?

R- I wouldn't have done it. If I didn't see that 10, 20kg go off then I would have said what for? Because I've seen in 2 days, 5kg go, and I just did the shakes, this does work. I've done other diets and its only after 3 weeks you see the results. So, this one topped the cake.

I- When you didn't see weight loss in other diets, did you stop because of that?

Participant transcripts

R- I did actually, not only was it not suitable for me because it was making me sick. It's just didn't work. The tablets didn't work because it made me sick and I wasn't seeing the results and I was going to the GP with that as well. I told them it wasn't working and they tried me on a few different ones. Then I said to my Dr, I want to lose weight, I've never been this big, I want to lose weight and see results. That's when she sent me to see you. I jumped on it and did it.

I- Do you think you would be still as motivated if we didn't offer bariatric surgery? Or do you think it's enough of a motivator to continue because you know you're going to get more help in the end?

R- If I do the surgery, I will see more results. Yes, I will do well without it, but I think I will go further with it.

I- Do you think knowing there is a possibility of surgery down the track, knowing that, that you are trying to do everything possible to get surgery in the end? Or is doing the VLED enough of a motivator to stick with it

R- It's hard to know. I am doing the shakes, but I feel I will just do better with the surgery.

I- So, you really want the surgery?

R- Yes, I really do. I thought about it and it's been almost a year. Yes, I am going to stick with the diet, but I will see better results with surgery.

I- Did you know that we offered surgery when you first came or is it something you learnt about whilst being here?

R- I think after 1 week I found out it was part of the program. But I had already started the diet and was doing it for 1 week. But I guess it was an additional motivator to add to.

I- How was your social life affected by the program?

R- It wasn't. My family understood that I wasn't allowed to have that, I had the shakes. My mum and dad being Italian, they would serve me pastas and bread etc, first course, second course, third course. I said to them look, I'm on this diet don't put anything in front of me; if you are going to put anything in front of me put the salad. And my mum knows. I just have my shake with my salad and water.

I- Do you think if you didn't have the support from mum and dad it would be a lot harder?

R- No not really, because I did diets in the past and they were in a different state and I was here. All my family lives in XXXX. I have friends who could see that I lost heaps, they look at photos and say look at you here and now look at you. Hearing that

makes me happy to hear that. Its motivating. I keep photos in my phone. I will never be that big again.

I- What skills do you think have helped you stay on plan?

R- I think believe in yourself and the information you gave me really helped. It taught me what to eat and what not to eat. It helped me understand and it helped my kids understand. Don’t eat this because this is what is going to happen to you if you do, you are going to put on. And they understand and they had put it in their heads that if you eat chips it’s not good for you, you are going to put on fat. I find that telling people what to eat and what not to eat, if you eat good things it’s going to be positive. You want to be thin, not big. You want long time health, not short time health. That’s what motivated me, I want to see a future. I think to tell people if you want to see the future, if you are very heavy, you have to do the positive not the negative. You have to eat properly and exercise. Watch what you eat and exercise and you will see good results.

I- Did you find having a diet that was very structured and clearly outlined what you needed to do helped?

R- Yes, after having the information in front of me, I put it in my head this is all you have to eat. I put it in my head and had to teach myself, this is the good and this is the bad. Keep the bad out! Change the way you buy food. Don’t keep the junk in the house. And I don’t have junk in the house. The kids ask where are the chips? and I say nope have fruit. And they understand now fruit is better than chips.

I- Was there any point in time where you felt like dropping out of the program?

R- No, I was never going to drop out. I like to be successful and talk to people and have counselling to motivate me even more. I don’t want to drop out. Even if I don’t do the surgery, I still want to come here and say – this is how I am going. I don’t want to stop.

I- If we told you, this is what you have to do for the rest of your life…

R- I’d do it. I’m happy to do it.

I- You’re feeling that motivated?

R- Yes

I- What about the cost of the products?

R- I found the Optislim was cheaper. The Opitfast was so expensive, even if you get it online it was costing more. As there is barely any difference between the brands I just bought that one. The products were affordable, if you think about when you do a shop and you buy; fruit, veggies, meat. All the things you are supposed to buy and then cut two of them out, its roughly the same price. The cost is still the same, it’s cheaper actually to the honest. The box lasts a month so it is cheaper.

Participant transcripts

I- And the VLED groups did you find it useful?

R- The first group I found useful. But I liked the one-on-one better because you could say more than in a group, you could talk more personally than in a group.
R- If I lived closer, I would have come every week, but because I live far away I can't. I didn't want to go to XXXX because they just started. Someone called me up from here and told me about it, and I said no, because they are just learning.

---Interview end---

23.2.2018
In-person interview ID: 3
Duration 65min

I- How did you end up coming to this service?

R- I was in contact with you guys 5-6years ago, maybe, but it wasn't in this building. I had about 6months were I was involved with a metabolic thing to do with RPA. But I didn't last long because it was very difficult for me to get here. Also, when I was coming I tended to do group therapy and I didn't particularly like that. I wasn't ready to share things with other people. The basically, 5 years ago now, then 12months ago mum called. And I begged my way back into the program and I started again in Jan last year.

I- Did you try to lose weight before starting the program?

R- Yeah, I had in 2010 or 11, I saw a dietitian at XXXX and she gave me advice on what to eat and what not to eat. This was different to what I had experienced previously. I also had some things going on in my life my mum broke her leg, and I had to look after her. At that time, I lost 60kg in 6months, I got sick of being good all the time, and over a number of years I put it all back on again.

I- Please expand, on the part where you said you were sick of being good?

R- Well basically, I was doing the paleo diet before anyone thought of it. Before it was popular. I literally read a few history books on how cave men ate, and it was basically that they hunted during the day, got a kill and then ate liver or something like that at night. So that's what I did. During the day I'd have no food and at night I would have a high protein meal. And I did, I stuck to that for like 6months. I was really into it, the lifestyle style and it really worked for me. And that's how I lost all the weight.

I- And what changed though? Was it "I'm do hungry", "I don't want to do this anymore", what was it?

R- I found that I hit a wall, I found it didn't matter what I did. You see I started exercising as well as doing the diet. I got down to about 150kg and I found no matter what I did I couldn't lose another kilo. I was lifting the weights, doing the bike and not a single kilo would budge.

Participant transcripts

I wasn't seeing any more results and it got to me. Why was I going to all this effort to lose weight when nothing was happening? So again, bad habits like drinking alcohol and soft drink started to happen again, just all these little cracks in the amour. I went from being very disciplined to not giving a crap basically. This happened slowly over time.

I- How did you feel when you were 150kg?

R- I felt great, fantastic. I felt I was riding a wave of euphoria for some of those months. But when I hit that wall, it was terrible. I felt like I was on a treadmill going nowhere. Pumping hard and going no were.

I- At any point did you think about what you needed to do to keep it off?

R- I had been told by many medical experts that I need to keep upping my exercise more and more. And I hated that, I was frustrated with that. I was already cheating with lifting more and also cardio. I got to a place where I thought ok if you are going to lose the next 4-50kg you are going to have to be doing 2hrs of cardio every day and I just didn't have it in me.

I- Did anyone ever tell you that weigh maintenance was ok?

R- To be honest with you, it was all pretty hands off. I had 2-3 contacts with this dietitian at XXXX, because I didn't have diabetes. So, mum basically talked my way in to gain a few sessions with them, but that was it. They were not prepared to see them often and only then I was only going to see a dietitian, and she was only going to talk to me about food. It was all hands off and really there was no holistic approach. It was basically do what you do. And I was ok with that, because I'm the person who has to not eat, and do the exercise. Maybe I could have used the advice possible, I don't know.

I- When you came here and found out you had to do the meal replacement shakes, what was your first thoughts about using them?

R- I was a little bit macho about it to be honest because I had previously been doing the whole starve yourself thing. Most days, I'd only have an orange or some blueberries and a coffee and that was it, until the evening. Then I'd have a steak. I thought meal replacements, who needs that? Then when I actually started doing it, I thought it was very helpful to me to get me through those hours. The thing is, when I was doing it the Palaeolithic way. I really got the "zest for life", that euphoria you get when fasting. So, I was doing it in a cheat way, I was riding it. I wasn't doing it in a mentally healthy way or a sustainable way- I found that out in the end anyway.

R- That zest for life thing, I had experienced it before. It wasn't a placebo from something I had read, I knew it was real. So, I really got into it. So, when it was do these meal replacements, I thought, well I don't really need to worry about that. But then I found it very helpful.

I- What made you change your mind about it?

R- I wanted to be seen to be compliant to the program that you guys wanted me to do. But also, my brother works for Isagenics and he gets me the product for free, he had previously told me about it too. He's like 65kg and never been heavy, and he totally believes in

Isagenics and belongs to that cult. He'd always come over and talk to me about different people he knows that are doing it. He'd tell me stories about a guy in New Zealand that lost 100kg and all this. And he kind of talked me into this. I thought I'd give it ago and play a straight bat. Do what's in front of me and not do it cynically.

I- Your comment about wanting to be seen to be compliant to the program, what did you mean by that?

R- Well, I wanted to give myself the best chance of losing weight first of all. If I was going to be here I wanted it to be honest. There was no point in wasting my time or your time if I wasn't going to do it properly. But also, I was told, if you are here for a few years and it's still not working we will consider putting you up for the surgery. If you have done the right things in the previous year. That meant I had to do it. Now I didn't want to do group therapy again, I was a bit stand-offish at the start. But I thought if that's what I have to do to keep the goal alive, well that's what I have to do. It wasn't that I wanted to be seen to be compliant, I wanted both. I wanted to say that I've done what you have asked, I've committed to the program and done it properly.

I- What you're saying is that you wanted to keep the choice for bariatric surgery open?

R- Yes definitely, even though that's my second option. I rather lose it the "natural way", I am not silly enough not to realise that I may need it in the future because my knees and my hips are pretty good at the moment still, but I am aware that that's not going to last forever. I better get stuck into losing the weight now before my body starts to breakdown.

I- What regime were you first prescribed when you started the program, for example how many shakes or scoops of protein?

R- I think it was like for the first few days or first week it was one meal replacement per day, then I went on to two shakes, and then a meal at night or a meal per day. The other issue with that is my medications, the steroids, I am pretty much guaranteed that I will be on steroids at least 2 times a year, or 5-6 times a year. When I am on big doses of oral steroids, it's like forget about it. Harm minimisation, and try not to eat the wrong thing, because I know I am going to be eating something. And that definitely throws a spanner in the works for me, in my ability to stick to the program. I know once the weather changes from summer to winter or winter to summer, that I am almost guaranteed to be taking steroids during that time. That does make it challenging.

I- To recap it was 2 shakes per day you're were doing and 1 meal at night. Did you add any extra protein?

R- I did used to add one of those milo poppers, made from skim milk and have one of those after my shake. I was using the Isagenics shakes, which I think is higher in protein anyway, than the ones you were using- I think it was Optifast?

I- Did XXXX say to do the meal? Or was that part of the Isagenics program?

R- I'm pretty sure that's what I was told to do. I can't really remember to be perfectly honest. But I think I was following what I was told.

Participant transcripts

I- How would you gauge your progress on that program?

R- Well, with me its 2 steps forward 1 step back. I'm pretty erratic with it because that tends to be my personality and also the drugs I take. It tends to be the way I am with most things anyway. On the one hand in the first 12months I started at 215kg and I got down to 200kg and I went back up to 211kg. I kind of feel like it wasn't a complete waste of time. But it's hard you know, some days I just give up. I still try, and it's important that I keep trying. I did want to get 90kg off by my brothers wedding.

R- Just because I lose the battle one day or one month I still feel like I don't want to go back to the way I was in 2011 and 2012 where I was not giving a shit. I feel like I have to stay in the fight. Mentally I have to stay in the fight. In terms of the score board, if you measure it by weight, it looks like I haven't done a lot. But mentally I feel I'm in a better place than I was, certainly in a better place than I was 3 years ago when I wasn't trying at all. So, I try to have a positive attitude about it.

I- Does that mean you think the program was over all successful, ok or average?

R- I would hesitate to criticise the program, the methodology was fine it was just a matter of me letting myself down a lot of the time, or the luckiness of the steroid thing you know, making life hard.

I- You mentioned a little bit about your personality and your medications as well, so those two factors held you back, were there any other things that held you back? Anything else that affected your ability to be able to stick to the plan?

R- There is definitely an element of, sometimes, not all the time, I do eat for emotional reasons.

I- So, are you talking about emotional eating, like eating because of your mood?

R- Well, yeah. There's no doubt about it when I am feeling bad about myself and I'm feeling depressed then I do eat to make myself feel better. That has gotten better though, but it still has happened, in the last 12months it has happened. I am trying to address that I have actually organised to see a psychologist in XXXX that specialises in weight disorders. I haven't had an appointment yet, but that's what he does. But he has agreed to see me.

I- What you're saying is mental health is a big barrier to adherence?

R- Yes

I- Would you agree that getting here is also a barrier? I know that you have mentioned this before.

R- Sometimes it's hard, like today, it's hard because I had asthma. Today it cost me $100 round trip in the Uber that my parents pay for me, so it's a commitment. Both in the effort

and money to get here. So, I try to take it as serious as I can and I don't want to waste any time.

I- When you were on the 2 shakes and meal, did you find hunger was an issue?

R- Well, I find it's a bit like when I was doing the paleo thing. The first few days are hard, but once you get through the first few days you get used to it and retrain your body. So, no, it's not, I don't find I respond to the hunger side of it that much. Its more other factors, like the mental health thing or steroid thing or pure dumb luck. Like friends come around and want to have pizza or want to get pissed and do BBQ and do fun stuff. And it's important to do that type of thing, but it's not good to do that all the time.

I- Did you find when you were losing weight it was motivating? So, the question is; if you weren't losing weight would you still trying to maintain the changes to your diet?

R- I would assume, well, it would be very very hard. That was part of the reason why I fell off the wagon the first time. I was still trying, still doing all the sacrifices, but I wasn't seeing any more results. I was used to losing 2kg a week for 6months, so once I stopped seeing that number go down it really crushed me.

I- Did you find it socially isolating to stick to the meal replacement? You kind of mentioned you have friends come over was that challenging?

R- Again, they come at night and that's when I have a meal. So, it doesn't really bother me. When my brother comes up and eats a pie in front of me I don't start hassling him to give me some of his pie. That's not really me.

I- So, it wasn't a barrier?

R- No, not really. That's not really my go.

I- What kind of skills do you think are really important to be able to stick to a program like this?

R- The main thing was, you don't need to be hungry, you can eat stuff that's not going to put the weight on you and fill you up. You can eat cauliflower, tomato and broccoli. At times I have eaten a metric shit tone of that stuff and I've eaten stupid amount of it and still lost weight. So, when I'm using my steroids that's the type of stuff I try to get into and I know I'm not going to be putting any weight on me. But then I guess it comes down to sticking to that and not eating the bad stuff. Not eating carbs really.

I-What you're saying is that people need a bit of nutrition knowledge to know what food they can and can't have or foods the can eat a lot of.

R- Sure, and I don't know what it's like for people who are normally obese, but when you are carrying around a whole extra person you can eat that stuff and still lose weight. But I found that when I was only carrying around half an extra person with me, that wasn't the case. And that was part of the battle that was hard. The things that worked for me were no longer working and that has hard for me.

Participant transcripts

I- How long was it after losing all that weight the first time, how long was it that you decided to give up? A couple of weeks, months?

R- Again I didn't really measure it, so I don't really know. I don't remember just all of a sudden giving up and hitting the fridge, it was more gradual than that. And then the really bad habits that I know are really bad for me didn't happen again till 2013- 2014 so maybe a 1-2years later of gradually losing interest and the will to do it. Yeah it wasn't like I suddenly hit the wall, it was more gradual than that.

I- What was your goal?

R- Well, I wanted to weight 2 figures. I wanted to weigh 90kg. I would have been great. Realistically, I know I am stocky, but according to the BMI I'd still be obese if I weighed 90kg. So that's what I want to be, a normally fat person, not an abnormally fat person. That's my goal.

I- Do you have the same 90kg goal now or has that changed?

R- When I was losing weight, it seemed realistic, it was realistic. I mean I was only 40-50kg off it, I was half way to my goal. The past 12months I have found it harder, it has made me question if that's a realistic goal. At the moment, I'd be honest, I'd just like to get down to 190kg so I can get on a plane, visit my brother and not have to worry about getting a stooge to push me around the airport in a wheel chair because I can't walk the distance. Even just that would be awesome.

I- **This time round with the shakes, what stopped that process?**

R- I definitely had some bad weeks. I don't know what it was, the bad weather maybe? But in the past 12months I have definitely been using the steroids a lot more. I know my lungs have definitely been weaker and I know this because I get up every day and blow into the peak flow meter. So, I know that my lungs are less strong. I had bad weeks from that, and bad weeks last year when I felt bad about myself, so eating cakes and stuff like that. I wouldn't normally do stuff like that.

I- **Feeling bad about yourself, was that triggered by something, was it your depression?**

R- A bit of both yes. A lot of it is the natural cycle… the thing is I have been diagnosed with depression, but I don't want to take the drugs. It doesn't happen to be often enough for me to want to take the drugs. Look when I see the psychologist I will keep an open mind about it, and we will probably talk about. And who knows I might end up taking it. But at the moment that's the decision that I've made 20years ago. They tried me on those things and I didn't like how I felt on them. I know also you have to take them regularly every day, at the same time and I'm not very good at doing that. So, in the end I made a decision that I'm better of struggling for a few days every few weeks, than, taking a pill every day.

I- When you are going though those phases have you ever though, that 'nup I'm not doing this anymore'. I'm not coming here, as in, coming to the hospital program?

Participant transcripts

R- No, not really. I definitely had the 'not sleeping Thursday night' because I'm going to get weighed the next morning.

I-Did you have a bit of anxiety?

R- Yeah. And that was not you guys putting me under pressure, that was me and my family putting me under pressure. My family are very supportive and great, but I think I set myself up for a fall or for a bit of anxiety because every time I got on the scales I would sent 7 people a group text and say look I've lost 3kg, so they all know. And I kind of feel they do have a right to know because they all chuck in money to help me get here, so they are invested. So, I view this place with a certain amount of trepidation, because that's the score board.

I- What made you stay then?

R- I want to live. I have nieces and nephews. I like life, I'm not an unhappy person most of the time. I want to see what is going to happen.

I- So, the goal is living?

R- And when I'm this big I am uncomfortable. When I am down to 190kg, I am not that uncomfortable. I can carry that weight pretty comfortability. But being the size I am now at 215kg I ache and I struggle… well like to broke my bed. For most of Jan it was broken, and it was such that to get off the bed I had to do like a Chinese acrobat style manoeuvre, like a flip thing to get off the bed. Because there wasn't anywhere where I could put my left leg! My family came over to watch me, because it was hilarious. My left leg probably looks like the incredible hulk right now from a month of doing a one-legged squat.

I- What did you think about the groups? I know you mentioned at the start there was a bit of resistance to groups, so what did you think in the end?

R- Well, I did quite a few of them with you guys and when I was here the previous times. I do have empathy for other people and for their struggles and what they are going through. There is a certain novelty to sitting in a room where you are the second smallest person, that's awesome. But to be honest with you, being selfish about it, I don't want to put my shit on other people. I don't want to take on other people's shit. I didn't find it that helpful. I'm pleased if other people do, but I find it more of a chore than a help.

I- And because of that feeling you had, did that stop you from coming from any of the sessions?

R- I mostly still came because I wanted to still get on the scale and I wanted to be able to say, look I've done everything that was asked of me. So that if and when I am a candidate for surgery I've done it all. I've done it properly. That was important to me, then the group itself.

I- If there was anything that you could change, would you recommend anything?

R- Not really.

I- How about the frequency of appointments?

R- Well, if I lived closer, sure that could be easier to attend more.

I- But would you find it useful to attend more?

R- I don't know. Basically, the money thing means I can only afford to come once a month. All the hospitals in my local area don't want to know me because I don't have diabetes. I am just glad you guys are willing to help me.

I- In the groups you kind of said you didn't want to share stories, what is that about for you? What comes up when you're asked to share your experience?

R- Well, I feel like the positives and negatives are about the same. The positive is; well I'm not the only one, you also ate that piece of cake last week when you felt terrible, that's cool. There is a certain amount of helpfulness that comes from a shared experience. But also sitting there for an hour, and listening to other people's things, it's like I have my own shit to deal with. I don't have the mental space to think about it. I'm emotional and stuff. Like I cry at watching TV ads sometimes, I have enough trouble staying positive about my own things. Like I said I don't want to lay my shit on other people and I don't want to take on theirs. I could see how it could be helpful for some people, but it's not for me. It's not that I didn't want to talk about my issues, but it's just a lot of energy and mental capacity to listen to other people. I have a limited amount of energy that I need to channel into getting through the rest of my day, like putting the fork down and not eating. If I started to think about that poor lady who can't afford Optifast, and here's me my brother gives it to me for free. If I dwell on that it fucks me up. It's just stuff like that.

---Interview end---

13.6.18
In person interview ID: 4
Duration 20mins

I- What are you doing for weight management at the moment?

R- Cutting down the meals to smaller ones and no takeaway, no sugary drinks like canned ones. Mainly H2O these days.

I- You haven't been doing the meal replacements?

R- No, I couldn't deal with the taste of them and the cost was a big factor as well. I'm doing ok without them anyway. Except for the last weigh-in, it didn't look so great, but prior to that I was doing ok without them.

I- **When was the last time you took them?**

R- Would have been last year sometime, 6 months ago. I didn't do any this year.

Participant transcripts

I- How did you feel about starting a meal replacement program at the beginning?

R- Well, I did it for a while so I went out and bought it and I opted for a cheaper brand and the taste wasn't nice. I had never heard of meal replacements before I came here. I heard of Jenny Craig, that you had to buy her tucker. But the cost was too expensive, even when I was working.

I- Describe the meal replacement diet you did

R- I cut out two meals each day. I didn't have breakfast or lunch. I bought the energy bars and the shakes, you know the weight loss bars. I had whatever was appropriate for that day.

I- How did you feel on the program?

R- I never felt energetic or anything like that since I had my back injury. I was dealing with it ok. The experience on the program was positive. I could walk further distances, that was a big plus. Mobility improved. I felt like I was losing weight, some of my clothes that were fairly tight felt was a little looser. You didn't feel hungry either, that was another positive.

I- You felt that you were losing weight?

R- Yeah, I was able to do more things and not be as tired.

I- Did you have any difficult days on the diet?

R- No, I didn't have any difficulties. No, I didn't fall off track. Felt like I did everything I was supposed to do.

I- Did you add any protein?

R- No, I didn't have protein or prescribed protein. I was told to have 2 and then 3 shakes, no extra protein.

I- How long did it take you to not feel hungry?

R- After a week or so, or 2 weeks. This felt very different to what I was used to. It made it a little easier to stick to it. I took the shakes for 4-5months. But I wasn't happy with the results because I weighed in and I had gained 5 kg. That was really depressing and I thought I did well with it too and losing weight. My weight seems to fluctuate quite a bit. You know the last time I weighed I had gained again. But I am still lower than the weight I was when I first started. I was 159kg to start with, now I am 153kg. While I've put on 5kg here and there, I am not as heavy as I was when I first started.

I- Could you pin point where those 5kg came from?

R- No idea, I mean as I say I was eating small meals, I've cut out all the things that could be a hinderance to me such as sugar. Coffee I've gone from 2.5 to 1, and I'm working on going to none. When you've done it at 2.5 [sugar teaspoons] all your life cutting back to 1, that's a big

thing. Like in the last 6months, I've been getting only 1 sugar and I thought that was going ok.

I- Do you think doing the meal replacements has helped you with the changes you've made now,or are the changes completely separate?

R- I think its separate. Like, I've spoken to people who have had surgery and they have said smaller meals are the go. Not necessarily the shakes, but smaller meals and everything in moderation.

I am still seriously considering surgery as an option. Like, I think the success of the surgery all comes down to you as a person. If you approach it with an open mind and positive attitude, you will do well. Just as long as you're not going to bull shit about it. Your fair dinkum about what you're doing, you can go from strength to strength. I really do believe that. Nothing comes easy, it's all hard work. But it you're prepared to do the yards, then you will reap the rewards

I- How was your social life affected by the program?

R- Well, I don't eat out anyway because I can't afford it. That's out for a start, that just doesn't happen. During family occasions, they knew so it wasn't a problem. They knew what I was doing and why I was doing it they were quite supportive.

I- What sort of skills do you need to do well on this program?

R- You need to be 100% committed, that's a must. If you're not you're wasting your time and the resources. Be confident and be sure that you are going to be able to do what is required. You have to discipline yourself.

I- Did you ever think about dropping out of the program early, and why?

R- No, well, I did intend to stay on the program longer. But it got to the point where my income went from doing some casual work to being on the doll. I mean, that's just what happens. It had to happen, it wasn't a choice.

I- Compared to your normal grocery bill, how did the cost of the program compare?

R- It was a bit more expensive. On average a fortnight I spend $100 on groceries, I do the specials and I don't go extravagant. You have to be savvy with your shopping.

I- How did you feel about the program being delivered in a group setting?

R- I did like it. I was one of the lightest people there in the group, there was some massive people there it made me look like a kinder-garden kid. Seeing people bigger than me put things into perspective, there are people out there worse off than me in terms of obesity. Although, I'm described as being morbidly obese. But looking at those people, I am nowhere near that big. It was motivating being in a group with those larger people.

R- Seeing that person who was close to 190kg, coming down to under 100kg was like wow. The one in the talk, on the last day. She was motivating, not bad for an old girl. She's a real inspiration to a lot of people, I would think. I'm sure it wasn't a bed of roses for her either. I think the group was very positive.

I- How about the frequency of appointments?

R- I think the appointment frequency was fair.

---Interview end---

Date 19.6.18
Phone interview ID:5
Duration 50min

I- At the moment are you doing anything for weight management?

R- I'm on the Saxenda and doing hydrotherapy. The food thing, that's touch and go, because of the Saxenda kind of helps with the hunger do you know what I mean? So, I'm not hungry and I don't feel like eating.

I- Are you following a diet plan?

R- No, I'm just eating less right now. I keep trying to do the shake thing for a couple days here and there, but I kind of finding it difficult to stick to for some reason, for now anyway.

I- Initially how did you feel about starting a meal replacement program?

R- I kind of didn't know what I was getting myself into. I was just like, I need to do something and I don't know how I was able to do it for those couple of months. I wasn't thinking of anything, I just knew I had to do something. It wasn't like I wanted to think about it too much, if I did think about it a lot, I probably wouldn't have done it. I rather just go and do it than think about it too much.

I- Had you heard of meal replacement products before?

R- Yeah, I heard about it on TV and I have done some of them years back, just to try, but I didn't like it. More because of the taste wise, I don't actually know what was in there.

I- So, because you had done it before, it wasn't a deterrent, considering you didn't like the taste?

R- No, it wasn't because I think when I did it the first time I was in a different frame of mind. It makes a huge difference.

Participant transcripts

I- Thinking back at when you did the diet program, could you describe what this experience was like for you?

R- I was on 5 shakes plus 9 scoops of protein powder on top of that. I felt like it was too much. But I did it. I'd get up have a shake, then the next one. Then from then on, I felt like the shakes were too much for me so I started substituting the bars and things like that so that's pretty much what I did. I had 3 shakes and like a bar or dessert. I tried to switch it up a bit because I was getting bored with it.

R- I kind of tried to switch it up with different flavours, to find the right flavour for me. Some of the flavours were a little too artificial for me. So, I got a sample pack that had all the different flavours, I went and tried different brands. It took me a little bit to start, because some of the things I had in one brand, and some of the things I had in another brand that I liked. So, there was a little bit of a trial and error.

R- I ended up using the Proslim for the shakes, Optifast bars like the chocolate bars. I'm not a chocolate person, but that one tasted the best. I'm more of a berry type person but I didn't like it too much, so I went back to chocolate because it tasted more like chocolate than the others. Then I ended up having the plain vanilla shakes because the other ones tasted a little too artificial for me. The vanilla was truer to flavour.

I- So, taste sounded like it was a huge driver for you?

R- Yeah it was.

I- Did you ever have a time where you didn't add protein?

R- No, I've always added the protein.

I- How was your hunger levels?

R- The first week or couple of days was like (laughs), like you're having withdrawals of having physical food in your mouth. To be able to chew foods. That was a bit of an obstacle for me there. Once I got into the swing of things, it felt like 5 shakes was too much. A couple of times I couldn't even finish them.

R- But then I had to deal with being outdoors and thinking oh no I didn't bring my shake. Then I had to create a routine, like drop a couple of bars in my car so if I couldn't get back in time for my shake. But then after a while I started bringing the shake with me. So, it was an obstacle to try an incorporate it into my daily life.

I- Sounds like you figured it out?

R- That became, well I wouldn't say naturally, it was like well if I have to do this I can't just leave the house and I want to eat, I have to take it with me. It kind of made sense. That kind of helped, instead of being out there feeling hungry and then eating the first thing you see. At least you had the bar there to like have it.

Participant transcripts

I- You said chewing was something that you missed in the first few weeks, but were you actually hungry?

R- First it was hunger for the first few days, obviously because you need a few days for the things to start kicking in. After that, well actually, I was eating a lot of salads as well, towards the end of the day and sometimes I'd put a small piece of tuna or chicken to munch on. And then I was looking for snacks, something crunchy to snack on. I was surfing the net for a replacement meal with crunch, something like that.

I- It sounds like the texture of food was a big thing for you?

R- I've always had a thing for crunchy things. Physically, I could always do with a crunchy snack somewhere, rather than an actual meal. And then I found rice crackers, like a seaweed Asian crackers. I thought I'd give that a go. I don't know sometimes things looked healthy, but they might not be. They could be high in salt, because they taste salty so they must have some salt in there. I didn't know exactly how much salt was in there, I don't really understand all that jargon. Then I kind of got tired of it I guess, I couldn't sustain it for that longer period.

R- What I ended up doing was, trying to do it for a few days until I couldn't take it anymore. Then I started again, and then a couple of days the week after. So, it was touch and go each time.

I- How long did you stay on the full program?

R- I think it was 2 months, at first it was the 2 weeks, then I came back, then I did another 2 weeks. Then after that the cracks started showing. Also, what didn't help me was everyone else around me what eating. I was looking at the shake thinking- come on guys. I think it's like a visual stimulation and smell, everyone is cooking all this good stuff, I'm like come on man. I was like going out saying, don't eat this next to me. I was getting cranky with the people next to me, and I couldn't have it. After a while I was feeling a little deprived. It's all to do with the mind, I guess at the end of the day. I wasn't completely done with it, but I was starting to. I didn't give up completely. Plus, I was doing 5-6 days a week at the hydrotherapy pool, which helped me a little bit.

R- After a while I stopped losing weight, but then I was losing measurements around my belly and stuff. My measurements came down, but not my weight for a bit. So, then I started on the shakes for a few days and then some days I go whole days without eating.

I- How did hydrotherapy help?

R- It helped in mobility and a bit of weight loss. It also helped me stick to the diet a little bit because I can't have a shake before going swimming because I'd regurgitate it. Then when I finished I would be famished, which is problematic because I found if I skipped meals my hunger would come like double. I just want to destroy everything in front of me, it's a self-taught thing you have to do to work out how to get through it. You have to work out the timing and planning of your meals around exercise to work out when to have it. See I was going there every day at the same time, routine is the thing you need to get for a while.

Participant transcripts

R- Then I started swimming for a while, counting my laps, I made sue I did a minimum of 20. The weight started to plateau for a while, then I started losing in measurements after that. Then I dropped again a few more kilos. So now I'm weight stable for the moment.

I- When you did the full meal replacement the first time, how much weight did you lose?

R- I think 15kg all up. My starting weight was 270kg. I weighed myself the other day now I'm 239kg.

I- Is there anything that stopped you from being able to stick to the program as you had planned?

R- Stress. Because I became an emotional eater any moment there was stress in my life my first action is to turn to food. That made the battle even harder. At some points that happened. I kind of worked out that I'd have to be kind of locked away in a room somewhere and have someone give me the shakes as I need it. It's the idea that I kind of need to be in a straightjacket and someone feed me as I need it. I know that's a bit extreme, but sometimes I feel like that's the only way it going to happen. I know it's unrealistic, but sometimes that's the way I feel. Do you have a program like that?

I- **No, we wouldn't lock people away**…

R- It would be ok with me. I know it's a drastic measure. But sometimes I feel that's the only way it's going to work for me. What's happening right now with me, is that I realised it only works if you want to do it. As opposed to other people telling me that I need to do it. It came to a point where I thought I need to do it and I am losing it slowly and steady. Also, one of the downfalls was timing, like how long it was going to take. Like thinking; oh my god, I've only lost this much and you get frustrated. You've done all this hard work and you've only lost a little bit. You feel like I've started at such a high weight, this small amount of weight loss is just a drop in the ocean.

R- So what I had to do was change my thinking, it's all a mental game we all have to go through. We tell ourselves, oh man this is too hard. You have to get over that side of the mind because it's like a nightmare. So basically, it's managing your expectations, like I want it to happen now. But that's not helpful. What started to happened to happen to me was, I kept saying to myself, I don't care if I just lose 20grams. It's all around rewiring your head about the way you think.

R- I stopped worrying about goals, like X amount of weight in a certain time. My goal is to just lose weight, that's it. When I make concrete goals there's too much pressure on me and then I fail. So now there's no expectations, no time lines. As long as I lose I lose and that's it.

I- What are your motivating factors to sticking to the meal replacement program?

R- Health wise, obviously. I was tired of being the way I was. I was tired of functioning as a human being. I just wanted to be able to just walk down the road. Just normal stuff, that was my goal. Another goal was to go overseas. I didn't want to go and not be able to do anything. That was another motivating factor. I think something nice happening to you is a motivating

factor. I did lose some just before going there. I was able to walk around and not die out. I was able to enjoy more whilst I was out.

R- For me I have to think about why I was doing it for example, for your health and everything, that's a given, but sometimes you also need something else. Whatever it takes to get you over the line. So now I am already planning my next trip, so that's my next motivation.

R- I had to depend on a lot of people when I was away, take me here take me there. Next time I want to be able to do it all myself without having to wait for someone to take me somewhere. For far too long I've been reliant on others to do things for me, which really hurt my self-esteem. I started to feel like I was useless. I started to think I want to do this for me and no one else.

R- People kept telling me do it for your family, do it for your mum etc but none of that worked. I had to do it for me. This was my driving factor. For once I wanted to live and experience life. I guess for a lot of people they don't see the light at the end of the tunnel and give up. I'm not looking for something unreachable, when you put goals on yourself that's unreachable that's when you fail. I had to learn these things.

R- Most people when they say they want to lose weight, they want it done yesterday. I figure it took so long to put on, it's going to take double the time to take it off. In saying that it's changed the way I think my mental state. Whatever the little bit that I have done, I should take that as a positive.

R- So basically, it's about shifting your focus on something else. Like going to the pool has not only helped me physically but also mentally because I've started making friends there and started some friendships. This was both cognitive and physical treatments without knowing. Before I never used to like going to the pool.

I- Why is that?

R- Because I felt people were looking at me a judging.

I- Sounds like you were feeling stigmatised. Have you ever felt like you were being stigmatised about your weight on other occasions?

R- I've always being a big dude. But I never really felt that pressure when I was younger because I was a bit of an extravert person. So, I would be looking for attention. Not until the last 10years did I feel stigmatised.

I- Do you think this affected your ability to stick to the program or attempt weight loss?

R- Yeah, well at the time the mind space, I was an angry dude. I used to confront people who I thought was starting at me, so then I was heading down the wrong path. That's when I started to not leave the house because I didn't want to hurt anyone or get into trouble. There was only one way for me, like getting arrested. So, I had to change that. It made me an angry person, and its hard to motivate yourself when you're in a bad mind space.

Participant transcripts

I- How was your social life affected by doing the meal replacement?

R- My social life was non-existent, besides from being around the house. I wasn't really going to any parties or anything like that. Every time I got invited I would chicken out because I didn't want to be made fun of. I used to have a decent social life, but not anymore, but once again its picking up again. Since I've more contact with other people and some of the older people. You see I stopped reaching out to people. Now I'm starting to get back into the mix and it's getting a little more now. Weight loss helped me think better about myself and when that happens I feel like I can be out. It's a chain reaction of events.

I- Have people commented to you about the use of the meal replacements and/or attending the program?

R- Not really, even healthy people wouldn't go near there. They would say "I would do that even if you paid me". It was not enough to deter me. It was never a reason why I fell off track.

I- How about the cost of the program?

R- Depending on the brand of the product some were more expensive than others. Obviously, Optifast was the most expensive, but I sampled a lot of them. Some of the Optifast stuff I wouldn't touch because it tasted like wood, and the chicken soup tasted like I was drinking saw dust. Then I tried the different brand and found some products I liked. Some of them it just had to squeeze my nose and get it down.

R- Overall the program cost much less than my grocery bill, I don't have to think about that one too much. It's not like you have to buy a whole bunch of different stuff to go with it.

I- How do you think the staff treated you at the clinic?

R- I think you guys were nice. The only thing if I had to point a finger at is maybe if you've never been overweight, you never know what we go through. Just be sensitive about things. I wouldn't expect you to know how it's like to be someone like me. So sometimes when you ask a smaller person to do something, it's not as easy for someone like me.

I- So, would you want staff members to be more sensitive about certain topics?

R- Well, I don't think it's something you can teach someone. Sometimes someone will just say "hey why don't you just climb over here?" something like that then I think – yeah right do you realise how big I am? They aren't thinking properly about what exactly they said. Sometimes people have different abilities to others. This stuff wouldn't make me go off the rails, but it's just something to think about. I think staff have to have empathy, but it's hard because you've never been in the other person's body. It's not something I'm angry about though.

R- To give you another example, I went to a clinic for bariatric surgery many years ago. I am guessing people who search bariatric surgery would be obese, well most of them anyway. But when I got there I couldn't even sit in my chair. I got there and thought, what kind of place is this? If you're not a big person you don't think like that. The clinic obviously didn't think

about the size of the person they see. What I liked about the obesity clinic is you have big chairs for big people. That makes sense.

R- Sometimes I watch infomercials on TV and they say they have lost so much weight, but none of them ever started off with as much weight as me. It's a bit weird like that. You never see people who have lost weight then go on to teach others. I mean that's too much really.

I- What about the groups?

R- You got to understand that we all walk in some sort of mental and physical prison. When we meet someone, who looks like us we are a bit standoffish, then once the ice is broken, then initially when you see a bunch of us together its very depressing, to be honest, because everyone is looking at each other thinking, I wonder how heavy he or she is? It's a natural comparison thing that we do. I bet it's the same if you get a bunch of fit people together, it would be a competition to see who could do what.

I- Some people have told me that they felt comfortable in the group, because they felt like "I'm not the only one". Do you agree or disagree?

R- I definitely agree because you can relate to whatever they say. It's always like that though, if you get a bunch of people in the room; big, small, black, white or yellow you're never going to be best friends in the beginning. It's not a big people thing, it's just a thing that people go through. That's when your character shines through.

R- For me I don't take things too seriously, I make jokes and all that. I mean I see big people out there that just look down and out all the time. I think I wish I could have the power to just snap them out of it. Hopefully, one day I could be an advocate that could just snap them out of it. It only takes one person who has been through something to be able to teach about that thing. It's a bit hard sometimes to listen to people who have just learnt some stuff from a book, with no self-experience. You can't buy that.

R- I met some guy in the group who didn't want to be in the group, he said 'you did the same thing I did, oh you're on the same medication' then we started talking. I think a lot of people are on their own, until they are in that group. Then they start finding out that there are others just like me. You know society is cruel people are always going to point at the person who is different to everyone else and once you start accepting those facts you'll be fine. Well, I have. I don't want to kill anyone anymore, because they are staring at me. But then I get upset when I see someone being rude to someone else. Especially if they don't know you, like they just want to make themselves feel better by pointing out someone else.

I- What about frequency of the appointments?

R- I didn't mind, the only thing I didn't like was the parking. We are big people, we can't park 2km down the road and walk 2km. These little things deter you and the obstacles that stop you from coming. And that's what got me eventually, it was just too far. I get there, I have to come there at least half an hour early and drive around like a crazy person and try to find one spot here and one spot there. Then you think, man, I have to make this appointment then you park anywhere and you come back and get a fine or pay for parking and it's not cheap. I don't understand why they aren't building a car park, are they building a car park?

I- Yes, they are. But you know, these things take time.

R- Yes, I know, and there's always some sort of road works and less and less parking spots available. It's just a nightmare. It's one thing driving to RPA and then finding parking on top of that. Actually, I met someone at the pool who also went there and said they were still going because they didn't know of anywhere else. They didn't know about XXXX.

R- At XXXX I see the dietitian, psychologist and doctor. So, it's great, I get the motivation from them and other people I talk to that's taking the same journey.

R- But you know your team is A+. You have to look at it like, you guys just want to help me so this is just me nit-picking.

---Interview end---

18.6.2018
Phone interview ID:6
Duration 36min

I- Are you doing anything for weight management at the moment?

R- I've just had bypass surgery and now I'm on the soft phase of the diet. I'm still having an Optifast in the morning and trying to keep the protein up for the healing stage of the surgery.

I- When you were initially told that this service included the use of meal replacements, how did you feel about starting a meal replacement program?

R- I hated it. I thought oh no how am I going to do this? Because you know I love food, food is life, food and eating is what you're born to do. And then you say to go on meal replacements I thought, I don't know if I can do this. But I had to, that was my motivation. I couldn't breathe I was as sick as anything and I just knew I had to do it.

I- So, you feel as if you got to the "end of the line?"

R- Exactly, yup.

I- Thinking back at when you did the VLED program, could you describe to me what your diet was like?

R- To start off with I had 4 shakes per day. That consisted of, I only like the Optifast and only like the coffee, because I love coffee. So, I had my Optifast with a teaspoon of coffee in it and I also had a scoop of Beneprotein in it, which I have to say was a life saver. Because without that Beneprotein you still get hungry. I have had the shakes without the Beneprotein and now that I have a smaller stomach its fine, but for those initial people that have to start off and have that extra-large stomach and I was one of them, and I could eat quite a big meal, I could eat quite a lot through the day, not big meals but consistent enough. Put it this way, I

could eat. I found with Beneprotein in it, it really curbed the appetite and I was full, and I could do it. But without out that it would have been a struggle and I wouldn't have done it.

I- How many scoops of protein did you put in each shake?

R- One scoop in each shake. I did it like this for 8months. Then I had a period where I ran out of protein, so I didn't have it. But I noticed I got hungry again, so I re-added it myself. Then when I got to the 10month mark I had to stop it all together and cut down the shakes as well. When I added the protein though using meat, I stopped the protein in the shakes. I started adding chicken and fish in my diet and cut down to 3 shakes and started adding more vegetables.

I- Did you find the food-based protein had the same effect as the Beneprotein or what it different?

R- It was different, it wasn't as filling. But by then because I had been dieting for so long it was sufficient. The protein powder was more filling. I've got to query the effects of the protein powder. You know I have that liver problem. I am in the process of seeing what has caused that because that's a long time to be on protein powders, you know 10months. I always knew I had a fatty liver. Did that contribute to my poor liver? I was told by my chemist that it could contribute to problems with urine, but we are in the process to see what caused my liver problems. Just me ruling out things.

I- The protein would not have caused liver problems. Sounds like you did the intensive phase for quite a while though, did you have any bad days?

R- No, the only time I broke. I started in May and my first break was Christmas. I went right through. At 7 months and I had a break at Christmas. That was for 3 days. I couldn't eat that much because my belly had shrunk, but I did enjoy all the things I did miss. You know when you go on a diet the first thing you miss is the sugar, and I was never a sweet person. That's what I couldn't work out. I thought I was going to crave Smiths Chips, but no, I wanted sugar, sugar, sugar. I sort of ate all the things I shouldn't, like chocolate nuts and those sorts of things. So that was my first break, my second break was on my birthday XXXX and I broke when my husband passed away in XXXX of this year and that was it until I had the surgery.

I- I'm sorry to hear about your husband passing away. Do you think emotional eating is what got you there?

R- Well, it was emotional drinking. I am usually not a big drinker, but on that day, I drank a full bottle of Baileys, and yes, I regretted it and will never do it again. You know I also had a few nibbles on things that was out, but that was it, it was all emotion.

I- What made you stick to the diet for so long?

R- My granddaughter. She's XXXX and I looked at a photo of me the Christmas before and I said I'm not going to see my granddaughter grow up. And it sort of stuck in my head: if I don't do anything, I am not going to be around for this little one. She's the going to be the only granddaughter I'll ever have. You know my daughter has XXXX, so it's a miracle she could even have a child. I've got three boys and one's handicap and he is never going to have

any children. The other two are definitely not interested in having children, they're in their late XXXX. I figured if I am only going to have this little one then I want to be here for her and she was my motivation. She lives with me, so whenever I felt like I wanted something extra, I would just say; come to nanny. I'd sit there and say I'm doing this for you. I'd just sit there and talk to her. Then when my husband passed I thought, well that only leaves me to be here for her. So that's when I thought, nup and you're having this surgery too.

R- At that point I was still undecided about surgery right up until he passed. Now I turned around and thought, well who is going to be here for my handicapped son? He's XXXX, but he is only functioning at the level of a 13-4year old. Who's going to watch my granddaughter? and that was my motivation.

I- They are very emotionally underpinned goals. How much weight did you lose just doing the meal replacements?

R- Over the 12months before surgery, when I started with you guys I was 180, but officially on your records I was 175kg. Pre bypass surgery I was 101kg and 3 weeks post now I am 95kg. I am only 10kg off my goal weight.

I- How has it changed your life?

R- I can move, I can breathe. I can go to the park with my granddaughter. I can't run with her, because of all the other things due to be previously being overweight has slowed me down. Now I can go for a walk with my granddaughter, go to the park and go to the shop. I went to the Easter Show for the first time in 15years and I got on a kiddy ride with her and it was so exhilarating. I thought, why didn't I do this with my children? I know they are all adults now and I did do it when they were children, but not in their teen years. I could have been running around with them and kicking foot balls and everything else. But I was too busy eating myself to death. Well, my big weight gain came after my mum passed and she was only 62. It spiralled after that. I didn't care about me after that, I just wanted to be with my mum. It was the little one who bought me back. She had me up all night last night. She got up at 1am and she wanted her nan. I had to sit up with her from 5.30am this morning because she wanted to go downstairs and play. I tried to tell her its dark outside and it's not play time.

I. At any time during the program, did the way you feel about yourself impact on your ability to stick to the meal replacement plan?

R- Of course, when you are big you get looked down on and that makes you feel bad. People say 'oh no, no', even my friends. It's amazing now that I have lost weight, I've lost friends. Because eating is social and if you can't eat you don't get asked to go places. You don't get asked to go to weekends away anymore because it's all eating and drinking. I've noticed that I only have one or two left now, all the other fake ones will still call and ask how you're going and say, 'Oh wow you're doing well now'.

I- Your describing feeling stigmatised because of your weight.

R. That's what it is, you are stigmatised, and you feel like you can't do it. Even the other day I went into the clinic and I saw some really big people and I thought please stick to it, I really

hope you do. Don't look at me like you shouldn't be here, because I was you. I felt like saying that to them. But I know as a big person, you don't want to hear that from a thinner person, because it's so god damn hard in the beginning. You don't see past that. You don't see that I could end up like that. It just sticks in your head, this is me, this is how it's going to be. I really don't know how I did it. It's a very hard taxing, long process. But for anyone trying to do it without Optifast and Beneprotein, I don't know how you would do it. I tried years ago with Prof XXXX and I tried a milk diet. I lost weight really quick, but it came back real, quick.

R- I think it did affect my ability to try weight loss because I thought; I don't have to justify anything to anyone else, like I am doing the work right now. Who are you to judge me? And that's kind of the downfall. You walk around thinking, it doesn't even matter if I am trying people are going to look down at me anyway. So, what's the point in trying? So, it did have some impact.

I- That must have been at least 15 years ago?

R- Yes it was, I had to have a full XXXX and they sent me to them to try and lose weight. And I did lose 30kg to have this operation, but then after that. I pulled through that and thought 'oh well'. Then my mum died and it all went downhill from there.

I- Did you need a lot of support to be able to stick to plan?

R- You have to have that one on one support. When you are big yourself conscious as it is. I can sit and talk to you fine, but if you put me in a room full of people nup, I can't do it. People think that I'm really confident. Even a Dr the other day at the hospital said, geeze XXXX you're a really strong woman. – No, I'm not. I'm on anti-depressants, I've got anxiety. It's not as bad since I lost weight, but I still have that anxiety. It never leaves you. And you definitely need that one on one, because crowds just don't do it. I've been to weight watchers, I've been to all those things and they just don't work. They are too impersonal. Here you are looked at as a singular person and it's a one on one, you are different people. Just because we are all obese it don't mean we are all the same. One thing we have in common is food, that's our evil. Other than that, we are all different and need to be treated that way, not as one big hoard of people who have trouble with food.

R- You get to know the staff personally as well and you get comfortable. Even though you don't see the same person every time or the Dr every time. But when you do, it's like I know you because we have been through this together. It's just personal.

I- There a degree of trust, would you say?

R- Yes exactly. It's like just after the surgery, I didn't care what the surgeons had to say I was too busy trying to get in touch with you guys. I didn't care what he thought because you become like our local GP, you know what we are like, you know our story. The surgeon only knows you for an hour.

I- Did you ever receive comments from others regarding you being on a meal replacement program?

Participant transcripts

R- Oh, I'm still getting negative comments from my family like 'oh stuff that'. Even how after the surgery 'what do you mean, you can't eat that, don't be ridiculous', 'oh surely you can eat this, just have some of that'. But when you finally lose weight its 'gee you're looking good'. Yes, because I'm sticking to it and not listening to you lot saying; 'Oh come on it's a party' and 'eat this, eat that'. It is very hard with family, family is your biggest sabotage.

I- When you get the negative comments, how did you respond?

R- I say because I don't want to be like this anymore. I want to be able to breathe. That was my main thing in the beginning, is I couldn't breathe and that really scared me. Because I knew I stopped smoking 20 years earlier, then I thought it was my heart and I went to Dr's and it wasn't my heart. Then I went to a lung specialist, who told me it was my weight crushing my lungs. I thought, how can I be crushing my lungs, I walk everywhere. Then I started using a power shopper when I went to the shops and I thought is this what you want for the rest of your life? Be one of those disabled people in a power shopper.

R- Now I walk into the shopping centre, wave to the ladies and they just can't believe it. That this women 12months ago couldn't even walk into the shopping centre to get the power shopper because I couldn't breathe. Now I'm a woman who can walk around the entire shopping centre.

I- What is a power shopper?

R- It's like a wheel chair or bike that has a basket that wheels you around the shops. I was so bad, I couldn't shop on my feet for that long. I couldn't breathe, plus my back and my legs and stuff, but yes, I couldn't breathe to walk around the shops.

R- I went through a big transformation. I went past one of the ladies the other day at the shopping centre. She was standing on the escalator going past and asked my son, how's your mum? And I was standing right beside him. I said I'm here. She couldn't even believe it was me.

I- Well, both you and your son look completely different.

R- Yes, I was blonde hair when I first started now I'm blue black. The only thing I don't like is the loose skin.

I- **Tell me about the loose skin…**

R- It is a problem. I had a shower the other day and I had to get my daughter to come and pack me with cream because just lifting a breast up to wash underneath, it tears. My skins underneath is just so fragile, it's so heavy. I lift up to wash, it's painful. I can't even have a mammogram, because just putting my breast up on the plates, it tears. I've got bulls balls down the bottom, which is the pouch at the bottom, and you can't even fit it into pants now. I should be in a size 14 pants but you need 18s just to fit this pouch in at the bottom and that's not even with the belly. That is really getting me down, you know I feel like all this and you still look shocking and that's without my turkey neck, I have my wobble going on there. But that doesn't worry me as much as that pouch does. If ever I can afford or if ever they are

going to do something for me, I will get that pouch done first and get that vaginal fistula fixed.

R- My daughter even had a look the other day, she said oh mum that looks so sore. I wake up at night when I roll over, and the skin gets stuck underneath you. But I can breathe, that's what I keep saying to myself.

I- How about the cost of the program? How did it compare to your weekly grocery bill?

R- It was hard to start off with. I had help from friends because I was on a disability pension. But when my husband passed, thank god I was at the end of my journey. I had to swap to cheaper shakes, I had to swap to Optislim because it's a lot cheaper. It is an Australian brand, but it does not fill you. I was just constantly hungry and thinking I am going to break. How can one brand be so different to the other? The taste, I even tried the low calorie Tony Ferguson, well that tasted like fish. I was on the normal Tony Ferguson a few years ago and I could do that one, but I found the only one I can drink is Optifast. I don't know if it has different stuff in it, but it fills you.

R- I had one this morning and it fill me till at least 1-2pm in the afternoon and then I have to drink water and coffee in between. I have to try to fit in a bit of yogurt around lunch time and have my small meal at night. I had my first mince meal last night and it was beautiful, curried mince and veggies, oh lovely!

R- That was the first bit of meat I had in ages, before that it was chicken, fish. It was very fine, but it was nice. The Optifast definitely, you have to have Optifast and Beneprotein.

I- What about the cost?

R- Well, my chemist was really good. If you go around Optifast is usually $55, we could get discounts through you guys, but unless you like chocolate or vanilla, those are the only ones you can get discounted for a 21 box. But I only like coffee flavour and they are like $55 for a 12 pack. Well, when you are on 4 a day it works out bloody expensive. So, my chemist was doing it for $40 a box and the Beneprotein was expensive too. It was like $33 a can and it only lasted 8 days and he'd do that at $25 for me. He'd do it for cost price, so I was really lucky that I had a good chemist. It cost me all the way through. If you can tolerate the Optislim its more value for money because you get 21 shakes, you get two of those boxes, you get 42 shakes at $55 or sometimes $50. If you are on a pension those two boxes would last you two weeks.

I- The Optifast was then costing you $100 a week?

R- Yes, one week it was $100, the next week $120.

I- How much was your grocery bills before the program?

R- Well, I just ate what the family ate. I didn't spend nothing on me. I buy myself 1-2 Dares milk drinks per day, which would cost $6 a day.

Participant transcripts

I- The family food bill would come to more than $120 per week?

R- Oh yes, it still does. I have a house full of adults.

I- Is there anything else you want to add?

R- I can't talk highly enough of the Optifast and Beneprotein. I could not have done it without that. I've done shake before, I've done low calorie diets and it's just not the same. That is your full nourishment. I could have even gone without the veggies. Well, I only had veggies once a day. And I know this is naughty and I could have lost more, but I had Chinese vegetables nearly every night with curry sauce or oyster sauce. We worked out, it would cost me $10 to order Chinese vegetables from the takeaway place every day, but if I went to buy vegetables it would cost ten times more. Because they have mushrooms, spinach, broccoli and onion. If you try to buy all that, fresh veggies the cost is unbelievable.

R- I didn't want to touch food, I didn't want to cook anything because I'd sniff it and want it. Now I can, and the boys are loving it because mum is back to cooking, I was a cook for work for 25 years. But at the time it was too hard, but now I don't even care about the food.

---Interview end---

Date 18.6.2018
Phone interview ID: 7
Duration 32min

I- Are you doing anything for weight management at the moment?

R- Yes, I am having Lite and Easy delivery, I chose the 1500kcal plan. My Dr told me to do that one. To be honest I don't actually get through all the food. I have a basket full of fruit that I haven't be able to get through. I am getting support from my Dr, I'm doing it for my health, she weighs me. I don't get too many pats on the head, but the last few times I've seen her it has kind of been dropping down. I'm moving house at the moment so it's been a bit traumatic, but she has been very supportive.

I- Traumatic? What do you mean?

Participant transcripts

R- Well, emotional. There's just so much to do and it's the home I'm moving out of, I moved into the unit when I was 20 with my parents and my sister. It was very new, we were the first ones there, it's almost 50years being there and so it is a bit traumatic leaving there. And everything that goes with it. The packing, the paper work.

I- How has the move impacted your diet?

R- There's so many things going on in my head and to attend to I lose focus, and when I lose focus I either don't eat or I eat the wrong things. But its more the emotional things, I mean you can't get it any easier than getting food delivered. I mean it's just there. You just put it in the fridge and take the food out every day. But some days I just lose focus and don't feel like it today, and so I'll just get a sandwich from down stairs.

R- Emotions are a big barrier for me, in the past I've seen a counsellor and I still keep in touch now for this sort of thing. But for weight reduction, I've barely ever had the enthusiasm to do too many things. When I was buying a unit and moving, you see I suffer from anxiety and depression, and whenever it kicks in together it's a nightmare and something that really suffers is my food intake.

I- When you were initially told that this service included the use of meal replacements, how did you feel about starting a meal replacement program?

R- Well, I had done Modifast many years ago and then it kind of fell apart, but when I went back the last time to do Optifast I thought, I can do this, it's not that long. I sort of went in with that mind set, but then after a while it was just like being on the Modifast again, and I would drink it and it would come straight back up. People say it tastes like a chocolate milkshake, but I know in my head, it's not a chocolate milkshake, it's Optifast. You know, you just can't fool yourself.

R- It didn't eventuate after that, I just got so bored and sick of it after that. I tried other things as well, after they took me off it. They put together a bit of meal plan for me, but by that time I think I lost all the enthusiasm.

I- You mentioned the taste was a big barrier, but did the emotional stuff at the same time influence your adherence to the program?

R- I can't remember I think things were going on as normal at the time. That wasn't happening then. I think it was just boredom. It's like the same thing when I have to have a colonoscopy, you have to drink the disgusting stuff for that. I was more anxious about drinking the stuff before the procedure than the actual procedure itself or the results afterwards. This was a similar sort of thing. I would drink the glass down and it would come straight back up again.

I- Sounds like the memory of Modifast was a barrier?

R- There's nothing wrong with it, you put up with it for a while. You think yeah, I can do this, I can do this. But then after a while you just think, this is awful. I think my previous experience with Modifast definitely did not help.

Participant transcripts

I- Do remember how long you did our program for?

R- I can't remember, I can't even remember how much weight I had lost. It gets to the point where I have lost so much weight during different times, I lose track. Every time I do something about weight reduction, I lose weight and I can lose a lot of weight but then I crash and burn and gradually put all the weight back on plus a little bit more. So, every time I go on a weight reduction thing the end result is that I am bigger than when I first started. It's a bit of a rollercoaster.

I- What you are describing is what we call weight cycling. Do you think that you gain skills each time you try a diet or do you think it hinders the next attempt?

R- I think overall you gain more skills because each time you try something you learn something new and then you learn what works better for you. You learn what's nice to eat, you know if you were just going to have a normal meal without thinking about weight you'd think oh no I can't have that because that's fattening. But when you're doing the Lite and Easy you kind of look at things and think, oh good I can have some of this. It's all weighed out and packaged for you, so it's all balanced I'm guessing there's obviously a formula they use. So, you can eat certain things, like popcorn. How many times have you gone to the doctor and said I eat popcorn and they say you shouldn't be having popcorn. Or a little chocolate muffin. I don't know if it is real chocolate or pretend chocolate, but it tastes really nice. But you wouldn't dream of eating that if you were just trying to lose weight yourself.

R- I think it's all portioned, you can still have certain things in a smaller way. Take for example nuts, it all comes portioned into smaller bags. Normally, if I was having nuts, I'd have a great big handful or bowlful and you don't need the bowlful. But that little packet you think well that was quite nice, that's all I needed. You do it for a while and concentrate on it. You see, if it's something that is given to you, you can only eat what's there. So, when you try to do it yourself, you buy a bag of nuts and I wouldn't try to portion them. It is much harder to do it on your own.

R- You see, even having food at home, normally I don't even think or worry about it. But the moment it pops into my head I think 'oh I have those biscuits in the cupboard when so and so was coming', that's all you think of until you go out and eat the packet of biscuits until its gone. It's just mind games.

I- How do you think weight cycling has affected your mental health?

R- You do, you lose yourself esteem you look in the mirror and think, oh I look like and elephant. You even get that type of trigger when your outside on the street. Just a few days ago I told two ladies off when I was shopping. You're just in that frame of mind, I was walking to the lift and the two of them were sitting there talking and looking at my stomach. I could see where their eyes where focused. I just walked right up to them and stood in front of them and I said you rude women. You are both quite rude aren't you. It didn't stop anything and I still felt bad that they had been looking at me but I felt maybe I have embarrassed them the way they embarrassed me and I don't mind that.

I- So, you have felt stigmatised by the women about your body size?

Participant transcripts

R- Yes, you do and people just look at you or nudge each other. You think with all this stuff going on about racism and ethnicity, I mean where I work is XXXX. So I am kind of in the middle of all this, all this stuff about Muslims or Jews, so I am kind of used to people being welcoming and understanding. But when you go out there it's not just people because of their religion or colour, its anyone who is different. I just happen to be someone who is overweight. People have no idea why people are the way they are, it's the same thing as people who look a little different, like people who have one arm. People just look at you and they stare and comment. Most time you just let it go, but then sometimes something inside you snaps and you just think no, say something to them.

I- How do you think these types of experiences has affected your ability to stick to a weight loss program?

R- Well, that's the whole thing it probably has some sort of effect because I get depressed. You go and you eat something like a bit of chocolate or something and they you're annoyed with yourself for doing it and then you think well I've done that, what does it matter if I have this now or that. And the more you do it the worse you feel about yourself, your kind of destroying yourself in a way.

I- What skills are important to being successful at losing weight do you think?

R- I'm not sure about skills, but I just have to keep focused to make sure I'm doing everything right. Keep taking the medication that I'm on. It all has to come together as a whole package, keep talking to people and don't spend too much time at home alone. All these things have to go along right and the moment something goes wrong you have to try to deal with it. I'm learning that with buying the unit. You have to do a big clean out and organise things from my parents and that's a bit traumatic. You have to deal with stuff like that and still try to stay on track.

I- How did you find the support from the staff at MOS?

R- The last time I was there I found it very disorganised. You sort of go in and tell people who you are, and I don't think they even knew who was meant to be seeing me. It kind of became a little confusing. You'd walk around one place and they would meet you there, and they sort of come out and really wouldn't know who was going to see you. You'd see the dietitian or doctor and go through things, then the next time you go the dietitians gone on maternity leave, so you'd see someone else. It got to the point where I thought, they really don't know what's going on here. I was in with a group of people and there was one girl there taking the group, but I really didn't feel like she was bringing anything to the group. She seemed to be all very new and didn't know much.

R- I think I had to get weighed and then I was taken to another room and walk here and there. I think I got so much exercise being there that day walking everywhere and for someone who is obese, by the time you get to where you need to be you're thinking geeze I really don't want to be here today.

R- That wasn't everyone though, people are nice and at reception XXXX. I've kind of known her for many years because I've gone there on and off for years. They are all very nice, but just the last time I went it all felt really disorganised.

Participant transcripts

I- How did you feel about being in a group as opposed to an individual setting?

R- I liked the group, it was quite good. There were a few people in the group that were quite good, you'd kind of share info with each other. But then there were others that I thought I didn't think they were quite following it, they would make comments like; I can do this and I still lost weight or do that and still lost weight. You know you're not really following the program, not that it had anything to do with me to comment on it. You sort of want to talk to people who have had problems. You don't want to be there with people saying how much they have cheated and think everything is fine. And they weren't losing weight. If you don't have the right mediator to bring out of people and to get them to talk about things, like some of them would say they don't have any issues. Well you kind of think, if you didn't have any issues, you wouldn't need to be here because being overweight is an issue in itself.

R- A couple of the women were quite good. We'd ask each other how do you do this, how do you cook that and they would give you good ideas. You'd learn off each other. You think thank god they have the same issues as me. They have difficulties eating this and eating that.

I- Did you ever receive positive or negative comments from others in regards to being on a meal replacement program?

R- They all thought it was great, they were every supportive because I was losing weight and they could see it. I mean it's really good when people comment like that, that you're looking good, and you knew from the scales. It was good feedback from the family and friends. I told a few friends and they were very supportive. They said if there is anything we can do to help you, so they were very supportive.

I- What about the cost?

R- I didn't, I am the sort of person who does the shopping online one time it may cost $100 or the next week it could cost $200. It would just depend because I would be stocking up on different things. I never really took too much notice, I just buy what I needed to buy. I know that's very cavalier, but I have never really had to worry about that sort of thing. I just thought I had to look after myself. So, it didn't matter if it was more or less, I never really compared it. I couldn't really compare it anyway because I never had a weekly or fortnightly bill. Now with Lite and Easy I do, it's not like I go do the grocery shopping every fortnight and have a budget. If I need it I buy it. It never bothered me what I had to spend on Optifast. I'm still earning money, maybe it would be different if I was retired. I'd probably have to budget better.

---Interview end---

Date: 25.6.18

Phone interview ID: 8

Duration: 33min

Participant transcripts

I- Are you doing anything for weight management at the moment?

R- No, well I suppose, I should say that I am watching my diet but I am not doing a very good job of it.

I- Is there a reason why you feel that way?

R- No, well, I don't know. I guess I feel that I've made a better effort in the past. I mean, I probably did a little better when I was using the shakes.

I- How did you feel about starting a meal replacement program?

R- I think apprehensive about how I would be able to manage it because I like to eat. Just the restrictions would be hard, I am not very good with sticking to plans. I was worried about the restrictions involved in it.

I- Are you generally not able to stick to plans generally or are you talking about just with diets?

R- I'd say generally in life, I am not able to. I make plans but I never follow it through to the end. I usually fade away after a while and give up.

I- Thinking back at when you did the VLED program, could you describe me what your diet was like?

R- I was doing 3 shakes. Breakfast was 1 shake with protein powder, lunch and dinner was a shake with protein powder with vegetables.

I- How long were you able to do that for?

R- I went really well for 6 weeks, then I struggled for about 3 weeks. Then I changed my evening meal to include meat with vegetables. I was still having the shakes, but I also included the meat.

I- Did you feel you were able to stick to it for longer doing it that way?

R- The last 3 weeks were really hard, I think I changed over to only having 1 shake in the morning after week 11.

I- What type of results did you get?

R- Well, really good. Except now, I have gone totally backwards. I lost 20kg, I got down to 130kg. I couldn't crack the 130kg though.

I- Did the weight loss spur you to keep going?

Participant transcripts

R- If I didn't lose weight, I probably wouldn't have kept going. Like I said to you the other day, I would never make a good vegetarian because I really missed having meat. That part I would continue to struggle with if I kept doing that. I have thought that I should try to do it again, because I did get good results and now I have gone backwards again.

I- What made you fall off track?

R- I think it was going on the cruise. The change of routine and maybe that wasn't the whole thing because when I got back from the cruise I still had trouble getting on track again. But then, I've also had the problems with depression and anxiety, and that hasn't helped. That's really affected motivation with everything and the osteoarthritis got really bad and that's had an effect as well.

I- Do you think tasting textures and flavours of food on the cruise had anything to do with it?

R- Possibly, I know when I was doing the program the biggest problem I had was the flavour and texture. Texture had a lot to do with it especially at week 7 or so, I really started craving different textures. I did try experimenting with a few different ways of cooking vegetables, which helped, but not hugely. The taste and texture, flavour and texture- well texture in particular.

I- What texture did you miss the most?

R- It was really the crunchy texture. One of the things I ended up doing was baking cabbage and it made it crispy. That was one thing that helped. It wasn't a great flavour, but it had the crunch. I found the recipe on the web somewhere and it was quite nice. Everything on the program seemed to be quite soft.

I- What about flavours?

R- I am not quite sure. It's been over 6 months I can't remember. I know people always think when your big you like a lot of sweets, but I am not like that. Actually, can you believe the other day a lady at the shops said that to me? She just kept looking at me.

I- Has that happened before?

R- Well, yes of course. In certain situations, like catching public transport I notice it a lot. Like catching a bus people look at you like don't sit next to me or if you are already sitting there they don't want to sit next to you because there is not enough room.

I- Do you think feeling stigmatised like this has affected your experience on this program or your ability to stick to the shake program?

R- No, I don't think so. I haven't really thought about it because it's there all the time.

Participant transcripts

I- Making the connection from what you said before, about suffering from depression. Do you think your depression is partly caused by the way you are treated by others or is it separate to this?

R- No, I think it separate to it, because a lot of it has to do with work and issues and changes to do with work. I am quite reclusive really. Now I don't go out much because I have trouble walking and stuff like that. But even before that I was quite reclusive anyway, I didn't go out much or socialise. I rather stay at home. Eleven years ago, I bought my own unit. When I bought this place, I even went out less. That might have been a combination of having a mortgage and not having enough money, but it was also my little nest and I loved staying home. Before I lived in flats with stairs and now I have a unit without any stairs so there isn't much exercise to get from my flat into the car. At one stage I was living on the 4th floor with no lift. So, at one stage I was doing a lot of incidental exercise.

I- Of the social life you do have, how was your social life affected by the program?

R- It did a little bit. I know I did go out a couple of time whilst doing it. I looked for a vegetarian option on the menu. I sometimes took my shakes out with me, but never to a restaurant.

I- What skills do you think are important to be able to stick to the diet plan?

R- I needed motivation and results. Getting the results and seeing the results, that kept me going. Early on the results were really good. When they started slowing down it was a lot harder. The weight loss was very motivating and a reward.

R- Having nutrition knowledge also helped. I would look at ingredients and nutrition panels on food when I was shopping. I did a lot of online searching for recipes, using the list of foods that I could and couldn't have. Then I would go looking for recipes that matched those foods and things I could do with them to make it more interesting. I think also going to the group sessions and talking with others was important. Talking to other people who were in the same boat as you were. I know a couple of times we shared recipes, and talking to others about how they are going. Just getting ideas from others about how they were managing it, like tips and tricks.

I- Do you think you need a lot of support to stick to the plan?

R- Yes, support and you need people around you to support you and not sabotage you. Like at work we still had work functions like morning teas and people would offer you things, if you said no, as long as they would back off. Occasionally you'd have someone who would try to push you to have something. They would say just have this and get back on track. But I know what as soon as I had something I'll lose it. It was good having people who weren't going to pressure you and would support you. So, I told my friends about it and they were supportive by not offering any food to me. They made sure that I kept on track.

I- Did you ever receive comments from others regarding you being on a meal replacement program?

R- No, there's so many people at work who have dietary requirements like coeliac disease, as soon as you say 'I can't have that' they leave you alone. You just become another person with a dietary requirement. There was support here.

I- How do you think the way the staff treated you at the clinic help or hindered your progress?

R- They were really helpful. It was only the person leading it really. She was really good and supportive. When you went down there the reception staff recognised you and said hello. There were certainly no issues there. XXXX was good, if you had any questions.

I- What do you think about the cost of the VLED program?

R- I don't think there was any real difference between my regular grocery bill and doing the program and I was doing Optifast. I think that was the most expensive, well there were certainly cheaper options. But that was available at the chemist near where I live. It was a discount chemist, that's what they had so that's what I got. I don't recall cost being an issue.

I- What about appointment frequency?

R- I think I went once a week. It wasn't an issue. But I work here at the XXXX so it was on XXXX so it wasn't hard for me to walk across XXXX. I didn't have to go out of my way.

I- Anything else you wanted to add?

The only thing is having a variety of recipes of different things you can do with vegetables would be a help. I had to go searching online to find ideas and get variety. So, getting some support that way would be really good. Maybe putting that in the booklet when we first start. You see I was searching out vegan recipes.

---Interview end---

Date: 25/6/18

Phone interview ID: 9

Duration: 25min

I- Are you doing anything for weight management at the moment?

R- I'm eating normally, but smaller portions. Healthy meals though.

Participant transcripts

I- When you were initially told that this service included the use of meal replacements, how did you feel about starting a meal replacement program?

R- I thought it was going to be a challenge. Coming from someone who abused their diet, but because I was so worried about my life, I welcomed the challenge. If this is what I have to do, well let's do it, I have to do it. I knew it wasn't going to be easy, but you know when you have your back to the wall and you have no other choice. You just have to do it.

I- What was making you feel like you had your back to the wall?

R- I was house bound, I wasn't able to go down the stairs. At the time I was living on the 3rd level of an apartment block and I couldn't do my shopping my weight had ballooned so much. Between the lymphedema, osteoarthritis and the bilateral knee osteoarthritis, I couldn't even breathe anymore. It was really bad, I said to myself I'm either going to die and give up on myself or I have to go and do something. Then when my social worker suggested a support worker who could take me to RPA, she asked me if I can get you help would you do it? I said yes. Then when I came to RPA and spoke to XXXX, so the support was there. Then I said to myself, I have all this help, all these people who are willing to help and my family. My family was really worried about me. You know I have been big all my life, but my daughter said to me you know you were able to move around, and now you can't what are you doing? I wasn't able to look after the grandchildren, so I did slowly with perseverance.

R- I spoke to XXXX every time things were going wrong and she helped me to deal with things. Between the support worker, the dietitian and all of you guys there at the hospital, I just kept going. When I could start seeing the weight coming off I could see the reward. I was feeling better, breathe a bit better, even with only a few kilos off.

R- There were times I just wanted to give up, but I kept going. There were times that I thought, I can't do this, I just can't. The craving where so bad, I was craving foods that normally I wouldn't have but I think because I knew I couldn't have it, I just wanted it. I kept going.

R- You know everyone has bad day occasionally, it's not a perfect process. Even though I stuck to it 99% of the time. On a bad day, I thought I'd had enough. Your mind plays tricks on you. I know my family were there supporting me and encouraging me and saying positive things, but then for some reason, well I felt like I was being made to do this. I had those negative thoughts- why should I be made to do this? After realising that eating like the old me made me feel off, it was not that everyone was making me do it, it was now my choice. I want to do it. You know and those thoughts don't last long, you have to pull yourself out of it, because you know it's not true.

I- What was your meal replacement prescription?

R- I was on 4 satchels per day, and vegetables for lunch and dinner, I did this for 12 weeks. After the 12 weeks I was still on 2 satchels per day and replaced one meal and I had extra vegetables. That was 9 months ago I had lost 40kg, now I think it's another 35kg. I still have a long way to go. But now I no longer use my walker or walking stick, unless it's a long distance. But at the moment I am managing on my own. Lots of rewards.

Participant transcripts

I- Sounds like your mobility has improved significantly?

R- Yes, my mobility, my breathing, even the pain. I'm not on any pain medications at the moment, I'm not even taking Panadol Osteo. Sometimes I still feel pain in the knee, but it is bearable. So, I don't take anything.

I- All this accomplishment with just weight loss- that's amazing!

R- I know, even my grandchildren say, Nona look at the way you are moving. You weren't able to do that months ago. You can see all these little changes, and you keep going because I never want to go back to 187kg never, ever!

R- And you know if I'm not careful I could. I could just eat the wrong foods I can put on weight, slowly, but I can. But if I can help it, I'd never go back to that weight, never ever!

I- What did you think affected your ability to stick to the diet program?

R- I found that with the Optifast, although I was using the satchels, I tried other products like Optislim. I found the taste of Optislim to be horrible, Optifast although it wasn't the greatest, but it was bearable. Enough so, that I could stick on for a while. The first 2-3 weeks I wanted to chuck it all in, it was really hard, but then slowly I could see the improvement. Each time the dietitian would weigh me there would be a few more kilos less, I could see I was getting a bit better. Then you look at the grand kids and get encouragement from the rest of the family. So, I just took each day as it came, I didn't look at the big picture, I just took it slow and kept going.

I- A few people have mentioned that . People have said any weight loss is still weight loss".

R- Yes, I'd agree with that. There were times were I only lost a little bit, but losing a little bit is better than not losing at all, so that's the way I had to look at it. Half a kilo today, half a kilo tomorrow. Look I'd recommend this program to anyone who is in the same situation as I was. The help you guys gave me, and give everyone else, I am sure we couldn't have done it without your help. And I still have a long way to go to reach my ideal weight, you know at least 60-70kg. It's better than a 100 something kilos I was before!

I- At any time during the program, did the way you feel about yourself impact on your ability to stick to the meal replacement plan?

R- The thing with me was, I never really gave myself a chance to lose weight previously. It's not that I've done diets and they didn't work, I hadn't ever tried to do anything. Five years prior to this occasion, I came to RPA to start this program. I had so many personal problems going on in my mind at the time. XXXX said to me unless you resolve all these other issues in your life, you are never going to be able to stick to this. I had to resolve all that depression and all the other things going on in my life. If I hadn't then, I wouldn't have been able to do it this time. When I came to you guys the second time, I knew I had the chance to do

something. My mind was free and ready to start and even with that I still found it hard to stick to for the first 3-4 weeks.

I- You never attempted weight loss before? Why is that?

R- You know why, it was because I was so energetic. I was faster than a slim girl and people used to comment on that all the time. They would say to me, my god your quick! I was big, but I could still move around and I was still going to work. Then all of a sudden, the lymphedema started kicking in and then extra fluid in my legs made me feel heavier, then the osteoarthritis in the knees and the carpal tunnel. And then it all come all at once, and the weight started climbing, but until then I could actually run. That's why I didn't really worry about the diet because I felt great and I didn't really care about the appearance. I just cared about feeling good and I felt good. It wasn't until 2009 that my health deteriorated and I couldn't handle it anymore. There were health issues and personal issues going on in my life and everything happened at once.

I- Was your social life affected by using the meal replacements at all?

R- My family understood, whenever I was with them, say I was going to my daughters for lunch, she would make me nice steamed vegetables because she knew I could have that with the meal replacement. So really you can still socialise, I know some times people try to tempt you and say "have some, have some". But no, my family knew not to do that. I had a lot of support even there, because they could see, they knew it was my last chance. If I didn't do this now, then I wouldn't live long and that's what they said to me. I knew that if I didn't do anything then my life would have been cut shorter. The first time I came there I didn't have a lot of support and I had to catch buses, with lymphedema, and get there myself, I don't think I was ready for it.

R- There was a lot of support from my family, a lot of support when I came to RPA and even my friends understood. If I couldn't have it, then I couldn't have it, that was it. My psychologist was also a big help with sticking to the Optifast, I still have her come to my home. She was a huge help.

I- Did you want bariatric surgery eventually, is that what motivated you to come here initially?

R- No, that only came later. When I first came there, I didn't even know it was an option for me. It was only until later I found out when people started talking about bariatric surgery. They explained that I had to go on to this program and come to us for a whole year, so yes bariatric surgery was only mentioned the second time I went there.

I- What do you think about the cost of the VLED program? Was it a problem?

R- Not really, because you still have to buy food. So instead of buying the $60 a week of groceries, you just buy Optifast. But my advantage was, and I didn't know at the time, that I had a package organised by social worker for people with disabilities. Through this they were covering my Optifast. I would buy it and I would give them a receipt and get the money back, I had about $20,000 in this package. Through this, I was even given a computer. But even if I didn't have this, I would have done it anyway, because you still have to buy food.

Participant transcripts

I- How about the way staff treated you at the service?

R- Of yes of course, the way they treat you, the way they encourage you, yes of course it helps. Obviously, it can affect you if someone puts you down for not losing weight, but that never happened to me. Even if I didn't lose weight, XXXX would say to me don't worry about this time, lets focus on next time doing this. It was just lovely and encouraging. I cannot complain.

I- In regards to how the VLED group sessions were delivered, how did you feel about the group sessions you attended?

R- I did the group once and then I did individual sessions. I was only offered groups once. That's when she prescribed the shakes according to our body weight.

I- How did you find the frequency of appointments and support provided at the service?

R- I didn't mind at all because I had a support worker who would bring me there. I was never late to appointments, always on time.

I- Is there any feedback you'd like to give or share with us?

R- All I would like to say is I'd recommend the program to anyone. There is so much support, but with the support you have to be willing to do this. People would be stupid not to take this help, people are out there willing to give their time and help. We should take it and embrace it and carry on.

---Interview end---

Date: 25.6.18

Phone interview ID:10

Duration: 27min

I- Are you doing anything for weight management at the moment?

R- I'm having meal replacement shakes and doing walking in the morning. I have a morning and lunch time shake and a normal dinner. Sometimes if I want a dessert I'll have another shake, so 2-3 per day.

I- Are you adding protein?

Participant transcripts

R- Yes, sometimes I have baby spinach and sometimes I have an egg. I haven't been using any supplements, I just crack a raw egg into it. I've never used the Beneprotein.

I- When you were initially told that this service included the use of meal replacements, how did you feel about starting a meal replacement program?

R- I wasn't happy, I didn't believe in it. To be honest, I didn't know how I was going to do it. But I am so glad that I did, because it's one of the best things that has ever happened to me. It helped me with my weight management. This program is one of the best things that I could have done, it changed my life.

I- Exploring the negative thoughts, why did you feel that way?

R- I tried them once before and I don't know, I didn't think, well I like food and the texture of food. So, I didn't think it was going to be for me. Now I really enjoy having the shakes.

I- What changed for you mentality about the food or the program?

R- About the food and the program. With the program I thought, I tried dieting and everything in the past and I'm just going to fail and when I started seeing the results using the shakes, and then got the confidence from you guys to start exercising, what things to eat and what things not to eat and stay away from, what to mix up like swapping from full cream milk to skim milk it kept kicking in and kicking in. And now it's changed everything.

R- I think the turning point was when I stuck by it for a week and then seeing the results was ridiculous, and I was like wow. So, I kept taking them, got used to them and now I enjoy them.

R- The weight loss was huge, I lost 7kg in the first week and that was without exercise. That was just with the shakes. It was a massive factor and the main reason I stuck with them, and it changed my whole thinking and mentally of it.

I- Did you ever have any bad days on the diet, tell me about it?

R- I have had bad days where I've gone and woken up and thought I don't want shakes today I just want to eat. It was a really bad day once where I just ate crap and I felt it that night the difference and it just felt bad on my stomach and though to the next day. The next day I thought I just have to get back into it and stick by it. I had to experience the negative side effects to remind myself not to do that.

R- It was like I was in self-destruct mode. It was like 'I'm over it blah blah blah'. And at the end of the day I didn't feel right. I actually felt ill and I never used to feel like that. I used to eat junk all day long in the past and feel ok, but now I can't it just made me feel sick. I just have to stick by it.

I- What made you get into the negative thought process in the first place?

Participant transcripts

R- In all honesty, I don’t know. It’s just one of those days, that I thought I’d just had enough. I’ll do and eat whatever I want. It was like a rebellious thought. Stick it to authority *laughs.

R- You know because I had my wife and everyone telling me to do it, and stick by it, and the more people told me, I had to stick by it, the less I wanted to do it. Then the next morning I woke up and thought, you’re an idiot. You’re doing well, just stick to your guns. I have to be around for my family.

I- What sort of results did you end up with?

R- So far, I’ve lost 30kg. I’ve been doing the shakes since November last year. I was doing 3 shakes per day and that’s all I was having in the beginning, but now I am actually eating and having 2 per day. I did the 3 shakes per day for about 3-4 months. I thought to myself I could still try to do that now, but what am I going to do at the end of it all. I have to start eating again, I don’t want to leave it to the end. So, I want to work on eating through it now. I can’t stay on the shakes forever, so I want to do the transitions slowly. Instead of doing, I’m on 3 shakes today and 2 tomorrow and then not knowing what to eat or what to do. So, I’m doing it slowly and listening to you guys. You guys are my key at the moment. You guys have helped with knowing what to eat, what not to eat, what to stay away from.

R- With the exercise as well, when I started this I could only do 1000 steps for a walk. Now I’m doing a minimum of 10,000 steps just in my morning walk. This is massive. When I first started I did one school block, I was so tired, I couldn’t breathe. Now I’m doing 7km in one morning walk. I feel great, now when I don’t get to walk I get the frustrated.

R- I had to have 3 weeks off because of my recent throat surgery and had to start again this morning. I was getting angry after 1 week because I couldn’t walk, so I was doing little walks, even though I wasn’t supposed to. Now I’ve started my 7km again. It feels good, I’m stuffed but it felt good. I know it’s going to take a while to get back into it again, but overall, I feel good. Look, I’m really in a happy place with exercise at the moment. The walking is working and I feel good doing it. I tried going to the gym once, but everyone looks at you. It’s really horrible.

I- That does sound horrible…

R- 100% it is, I used to see people look at me when I was out in public and make snide comments all the time, it was horrible. It felt like I was going to go through depression again from it. That shits, like, horrible- that’s the only word to describe it.

I- Did you feel welcomed at the clinic?

R- I felt welcomed when I saw people who were in the same situation to me. I knew there was no judgement. I feel great going down there all the time now. It’s like a boost in my confidence each time I go into the clinic. I don’t weigh myself or anything at home, except at my appointments with you guys.

R- It’s reinforcing each time I go there to see the results on the scale. Each time I go there I know I’ve pushed, I make sure I do everything right the two weeks leading up to my appointment. It keeps me focused and on target. I wouldn’t be where I am now without that

program. I'd probably be bigger. I know without the program I wouldn't have done anything. This is the best thing that's ever happened to me.

I- Who asked for the referral?

R- My gastroenterologist wanted me to come here. They said all the problems with my stomach was related to weight. He was the one who pushed me and set up the interview to see you guys and it is fixing the problems. I'm not 100% but I am much better. I've got GORDs, like bad reflux disease. With the weight loss I've actually cut down on 2 of the tablets I was on for it. My medication has been cut in half. My gastro said if I continue to lose weight, I'd probably be cleared of GORD.

I- What were your main drivers for sticking to the plan, was your daughter and disease control?

R- My wife and my daughter were my main motivators and then getting rid of the reflux. My reflux was so bad I couldn't even have a glass of water without regurgitating it back up. So hopefully with a bit more weight loss I'll be cleared of it. My other motivators are I just want to be healthy, as simple as that. I want to get out there and be able to ride a bike and play sports. You have to do it for yourself and you have to do it for your daughter. My daughter is 3, I'm scared that I wasn't going to be around if I stayed on the track I was on.

R- You know I lost both of my parents when I was young, I don't want that for my family. It's like a vicious circle for my family, my mum and her sister died young. I don't want that. I want to be as healthy as I can be. I want to change the pattern.

I- What about your social life, how did the shakes affect relationships?

R- In the beginning it was a little hard. My mates would eat, because you know they are all big eaters and you know I didn't want to be there because I didn't want to get tempted. But now because I know what I can eat going out doesn't bother me anymore. I still go out and have a good time.

R- I've learnt what I can order and have. It's pretty good, I can go out to social events and handle it. I can go to a party and go nowhere near the table, so I don't get cravings or eat because I'm bored. Some times I've even taken with me.

I- So, some skills you have developed are around food, planning, do you think there are any other skills you needed to have?

R- The skills I needed was what to eat, what not to eat, the portion size and social meetings, for example where to sit.

I- How did you think the staff treated you at MOS? Did this help or hinder your progress on the program?

R- What you guys do make a 100% difference in the results I get. The reports I get from you guys is very important to me, without you guys I wouldn't know how to handle myself during

a social event. I used to be happy to eat when I'm bored, sit near the food, it's all changed now.

I- Was it the groups or individual sessions that helped you?

R- Both, but more so the individual sessions. Group sessions were also great because I got to talk with other people hear what they were going through and what do to. So that was really helpful as well.

I- Did you ever get comments from others about doing the shakes?

R- Most people say it's good, they say you do what you got to do, I can see you have lost weight. Just keep doing what you have to do. Other say it's all in your head man just eat better. I just tell them, look I know it works because I am living proof that it works- simple. Their comments never stop me, if anything it drives me even more to try harder. I don't pay attention to their negativity, I'm a positive person.

I- What about the cost of the program?

R- I think the cost is pretty fair. I think the man shakes are a little bit expensive but compared to if I was buying food for myself, I'd probably buy all the wrong things and spend just the same amount of money. I think it's fine. People say, but you spend $120 a month, yeah but if I was eating junk food or takeaway I could easily spend that in month.

R- You pay $120 you get 4 bags of shakes, each bag is a week's worth, that's the Man Shakes. The Man Shakes are high in protein, and the sugar content is 10grams less sugar than Optislim. I think the taste and texture of this brand is pretty good. They also have good flavours like chocolate, strawberry, vanilla, banana, caramel and choc mint. There's a large variety of flavours and they also have Man Bars. I order it online, I think they have also started selling it at Coles. I'm on the Man Shake website, looks like they have been starting to sell it at Coles.

I- How about turning up to appointments and the frequency?

R- I think it's great, at the beginning I was turning up every 2 weeks then I went to once a month because I was doing so well. I think there was a couple of weeks where it was a bad, couple of week in terms of mental health, and I was told I can always come back to every 2 weeks if I wanted. Other than that, it's been fine.

I- Anything else to add?

R- I just want to say, from the bottom of my heart, I wouldn't be where I am today without you guys. It is tough doing the plan, but with the support from you guys it makes it easier.

---Interview end---

Participant transcripts

Date: 2.7.18

Phone interview ID:11

Duration: 20min

I- When you were initially told that this service included the use of meal replacements, how did you feel about starting a meal replacement program?

R- Well, I wasn't told to do it, I chose to do it when I was in XXXX hospital. I spoke to the dietitian there and started it 4 days before I was discharged. They referred me then to the Metabolism and Obesity Service, but I had done a full 12-weeks before I got there.

I- How did you know to ask about doing the meal replacements in hospital? Is it something that you had heard of before?

R- My sister has used it before, we had talked about using it before. While I was in hospital my appetite was suppressed because I had a bacterial infection. So, we thought, well we might as well start it now. My weight had ballooned up. I was a size 32 pants, size 28 top and now I'm in the 20's.

I- How much weight did you lose all together?

R- I started at 123.4kg, that was the Saturday before I started the Opitfast and I got down to 94kg. Last weigh-in, I was 111kg, I had chocolate at Easter and that started the sugar cravings. Then the dietitian I saw there said to go on full meals for a while, and that seemed to get rid of the cravings. But now I am full on Optifast again.

I- Overall how do you think your experience has been on the meal replacements?

R- I find it no problem. It really bugs my sister because she has so much trouble doing it. But for me, I've had controlled portions for years because I don't cook. I'm used to small meals. My problem is the sweets. Keeping on the Optifast shakes, bars and soups works really well for me.

I- How many are you on per day?

R- 4 per day. I have the vegetables on the side cooked in low salt chicken stock. To keep it interesting.

I- You said you found it easy, why do you think that is the case?

R- I think, I find it easy because my meals have been controlled portions for so long. I am not a big eater, I'm a sweet eater and if they are not in the house. I have mobility problems, so I can't go shopping. I do most of my shopping online and I don't put them in my order. So, if they are not in the house, I don't eat them.

I- So, you have removed the temptation?

Participant transcripts

R- Yes, so all I have is the Optifast and the soup. My sister comes, she is looking after grandkids this year and she comes on the weekend for time away. She brings her own food and takes her own food. See living by myself I think is good, I just have to do the shopping for myself and I don't need to keep food in the house.

I- What happens if you have a bad day?

R- That's going to be the problem, when I go off it. The last weigh in I was 111kg, I want to get to at least 70kg before I come off it. That will be the problem going on to regular meals then, but that will be a year or so from now. But I am not going to concentrate on that yet, not until I reach at least 70kg or 65kg. I'll be happy with that. My health problems will be so much better. I'll be still technically overweight, but at my age I don't want a lot of flapping.

I- What do you think affects your ability to stick to the diet?

R- Well, that's all that's there. There is no other choice. That's all that's in the house. Nothing else is going to affect it.

I- Do social occasions ever slip you up?

R- Well, that's going to be a problem in August because it's XXXX. I'll have to discuss that with the dietitians at MOS. I have an appointment there in early August, I might just have to go off it for one day and celebrate the day. XXXX is a big mile stone.

I- What type of results do you find motivating? For example, if you didn't lose weight but had better health would you still do the diet?

R- The way I am thinking and feeling now yes, but if I was feeling this good before I started probably not. I know the results, I'm a chronic severe asthmatic, diabetes 2, kidney function isn't that good, osteoarthritis in the back and knees, sleep apnoea and all these things have improved since doing the diet from last year. All of these medical problems will continue to improve if I just keep going. So that's why I want to keep going. Not having that knowledge before I started, maybe, I wouldn't have done it.

I- What there anything else that was motivating?

R- Once I got going on the plan and saw it working the major motivation was my health. At XXXX with the problems I've got, I told my sister once I'd live to 100. There was once the doctors told my mother I wouldn't live to go to school, my asthma was that bad.
So, I think keeping as healthy as I can, due to age, is my main motivation.

I- At any time during the program, did the way you feel about yourself impact on your ability to stick to the meal replacement plan?

R- No. I am happy with myself, even when I was my fattest. It didn't bother me. I am not very social. The people who do know me, take me as I am. I've done everything I've wanted to do. The width just never bothered. It doesn't really impact on me, I'm not very observant. I tend to stick in my little corner and potter around with what I'm doing. I don't pay attention

to what people are saying or doing, or if they are giving little stares. If you don't notice it can't affect you.

The only thing that may have stopped me, if anything, was financially it was a bit tight. It was a bit difficult to buy vegetables with the shakes to make up the meals, and buy the protein on top of that.

I- What skills do you think are important to be able to stick to the diet plan?

R- I don't know. It's just going so well, it's not even a matter of "sticking to it". It's just like this is what I am doing and this is it. It's a matter of fact. I get up in the morning and prepare the 4 shakes, drink one and put the rest in the fridge or pull out the bars if I am having bars that day. So, then I put them out of the fridge, as I'm hungry, and that's it.

I- People have said that nutrition knowledge, reading panels etc was useful, did anything like that help?

R- Oh yes, when I was doing the meals with Optifast, I was buying YouFoodz, they have a range of about 4-5 meals that have the right amount of vegetables and the right amount of carbs that you can have over the week for dinners. So that was handy having that knowledge, which I found on the little pamphlet that's in the little box. All their allowable vegetables are there, it's just a matter of buying them. Having the list of allowable foods was helpful.

I- Did you ever feel like dropping out of the program?

R- No. I had the sugar cravings. But I still wanted to do it, it was more like how to I get around this? How do I get back online? And it did work, what the dietitian told me to do to go on full meals for a while. That helped.

I- How about support, did you need a lot of support?

R- Mainly my sister, I'm not a good cook, she usually comes and cooks the soup for me for the week. I help her divvy this up, put a couple in the fridge and the rest in the freezer. I just pull it out and heat it up in the microwave.

I- Anything else you want to mention?

It's been very useful. I think if I just did the Optifast without being referred to MOS, I would have had more problems. Having the people there to talk to every month or so, that was very helpful. For example, they gave me a print out of how many calories and how many carbs I should be having for the meals, so then I could choose my meals within that range.

-Interview end-

Date: 2/7/18

Phone interview ID:12

Participant transcripts

Duration: 29min

I- What are you currently doing for weight management?

R- I am still doing the meal replacement program. I am having one meal replacement at lunch time, oats for breakfast and in the evening, I have protein, which is typically chicken.

I- How long have you been doing that?

R- I have been doing this for 6-weeks. I started the program last year, but towards the end of the year, I stopped. I got discouraged with it. I had a misunderstanding that I was not going to be eligible for bariatric surgery because I had depression, so I put on a bit of weight again. I still managed to keep of 10kg of what I had lost. But now I have started back up again.

I- When you were initially told that this service included the use of meal replacements, how did you feel about starting a meal replacement program?

R- I kind of thought, that's what would happen. I knew I would have to be on a diet of some kind. But I didn't realise it was going to be 3 meals per day, so that was a bit of a surprise. But it wasn't too bad of a surprise.

I- Did you know what meal replacements where or had you tried them in the past?

R- I did know what they were. I had done meal replacements when I was 18. I did 2 meal replacements per day and had lost 7 stone. It was nearly 35 years ago, right at the beginning when they first came out.

I- So, it sounds like you have had experience dieting before. How did you feel about the concept of dieting again? Was that discouraging knowing that you had to do another diet?

R- No, I knew that this is what I needed to do. I am very overweight or obese and I needed to do this to get into the program. I watched that TV show called my 600 pound body or something like that, on Foxtel and they didn't do meal replacements. They just did a very strict diet. So, I thought for me to lose weight I would have to do some sort of strict diet.

I- It sounds like you had a 'matter of fact' attitude that simply this is what you need to do, would you say that was the case?

R- No, not really. Diets are hard work. I knew that was going to happen. I didn't know it was going to be substituting 3 whole meals, that was kind of a shock.

I- Tell me, when you were doing the 3 meal replacements per day how did you find it?

R- It was a bit of a difficulty at first just getting into the habit, trying to prepare meals and trying to do the bowls of vegetables. You know making soups to keep in the fridge and stuff

like that, it took a bit of practice. It was ok for the first 4 weeks or so, but then I started missing things. I'm kind of a carboholic, I like bread and I like cereal and I missed that, I also missed fruit. I started to not stick to it as well after 3-4 weeks.

I- So, you tended to fall off track during the full meal replacement... so when you had difficult days, what did that look like?

R- I snacked. I tried not to have stuff in the house. I live by myself so it makes it a little bit easier, but I know how to cook, so I can make stuff. So that was a little bit difficult, so I had to get rid of all that. If I was out and about I'd pass a shop and pick up something. On really bad days I'd get takeaway or home delivery.

I- So how were your hunger levels like then on the plan?

R- The hunger did go away. It was just missing food that I was used to and I started to get bored. When I cook regular meals, I like to do different things all the time. So, I got bored having the same thing all the time. Like the vanilla and chocolate shake I didn't like, so that means I could only use strawberry, but then I started to get bored with it and there's only so many ways you can prepare vegetables!

I- So, it sounds like you found it a boring ?

R-Yes, especially if you're only sticking to one flavour. Yeah well, I am not a coffee person so the coffee ones where out and the chocolate ones didn't seem to agree with me. I'm also lactose intolerant or lactose free so that was a bit difficult as well.

I- Have you been helped out with that?

R- Yes, one of the ladies gave me a name of a different one, I still haven't found it yet. I can't remember the name.

I- What did you think affected your ability to stick to the diet program?

R- It was good to see the scales drop that really helped. I have a history of abuse as my background so it's not easy for me to accept compliments. So, when I started to lose weight it threw me a bit. I found I was sabotaging that. I tried to address it with my psychologist, but she really did not quite understand what I was trying to say. That was a bit discouraging too, because you know when people comment they say "wow you're looking so good" for me it was, I didn't know how to cope with it. I ended up sabotaging myself. I'm an emotional eater, it's something I am also trying to address because that became a problem. Apart from missing food, I didn't cope with the compliments I was getting from people.

I- Has that been addressed? Has someone helped you with this?

R- I've discussed it with the psychologist and I am going to ring up a few psychologists she recommended that deal with emotional eating. I am going to see if I can see one of those, but finances can be a bit of a problem. But I'm an XXXX so I have access to Closing the Gap, so I'm going to see if they can pay for the gap of seeing the specialists.

Participant transcripts

I- Yes, well a lot of people do suffer from similar things to you related to trauma and abuse. There is a degree of vulnerability associated with weight loss that needs to be addressed and skills developed on how to cope with these feelings. Its great you are going to see a new psychologist. It's good that you recognised this.

R- Yes, well I did try to talk to my psychologist about it and she didn't quite understand and I don't know if it's her background or whatever. But the suggestions she kept giving me were inappropriate. So, I decided I need to find another psychologist, which I am in the process of. I'm hoping if I find one that deals with emotional eating, I can bring it up with them and maybe they will help. Possibly after I get through that I will look for one closer to home, because the one I'm looking at is over at XXXX.

I- So when you were achieving weight loss, do you think seeing results is an important motivating factor to continuing on the program?

R- Yes and no. Yes, it does encourage you and you think oh ok I lost weight, each time you went and got weighed. It helped to know that I'm losing weight and not putting on weight. But then like I told you I started getting compliments and I started to sabotage myself. It helped in one respect but it didn't in others.

I- If you weren't losing weight, but you were getting health benefits do you think you would have continued?

R- I'm not sure, I don't know. I guess how I see it, is when you go on diets it's to lose weight. The benefits come into it but it's not what you're looking for, what you are looking for is the loss in weight. To say I would continue because of the health benefits but not lose weight, I don't know.

I- Can you pin point any other motivating factors that helped you keep going other than weight loss?

R- I want to do the surgery and I want to get healthy. My health has been really bad in the past year. Now I have rheumatoid arthritis and other health issues that I never had and it's a consequence of me being morbidly obese. That's the reason why I sought you guys out. I need to do something otherwise I'm going to be housebound because I am in so much pain.

I- Did you know we could offer bariatric surgery before you came?

R- Yes. Well, I wasn't sure that that's what I wanted, but I wanted to be a part of a program that helped. I don't find losing weight on my own helpful because I need to be accountable. If I am not accountable I don't tend to stick to it. The accountability of going in every week, I found that helpful, knowing that someone was keeping an eye on me. It was more the encouragement you know when they tell you to keep going, you're doing it right and then help you with things like overcoming cravings.

I- At any time during the program, did the way you feel about yourself impact on your ability to stick to the meal replacement plan?

Participant transcripts

R- When I was going the group education the 6 weeks leading up to the bariatric talk. XXXX was talking about depression and I suffer from chronic depression. I misunderstood, but at the time I thought I was going to be ruled out of surgery because of that. Depression is part of me and has been a part of my life for so long. Hearing that, I was very discouraged that I was going to be ruled out. But it was just a misunderstanding I guess.

I- Were there any other times when you felt discouraged?

R- Coming in that far was a real chore and expense as well. But I kept it up as much as I could. I managed to get some support financially, which kept me going for the first couple of months. The doing the education thing I managed to get extra funding for that. I have a car now so I can get myself in there, but it still takes me a long time to get there. It takes 1 to 2hrs to drive in to get to RPA and financially petrol and parking its tough. I can't park in the parking station where you get a cheaper rate because I can't walk that far. So financially its $20 parking on top of petrol.

I- So, it does become very expensive to come in each time?

R- Yes, well I've worked it out so I could come in once a month, at a stretch once a fortnight. But once a month I can afford that.

I- What skills do you think are important to be able to stick to the diet plan?

R- You need to be able to have a good knowledge of cooking vegetables. You know some people don't cook, but I was very creative with those. You know being able to cook them in different ways, really helped. Others have found it difficult because they don't cook and don't know that you can mix certain vegetables together. That in itself is helpful to know, so recipes. I know you have a couple in the book, but a recipe book containing a range of recipes would be wonderful.

R- Hmm let me think of other skills you need…being able to gain motivation and use willpower. You need to have some degree of willpower to cope with this, because you know I struggle with this at times. I don't know how you would motivate someone or help someone with their willpower. That's something that I struggle with this at times.

I- I think if someone could invent a pill with motivation and will power, they would be a billionaire.

R- Very true! But you are asking about skills, so I keep hearing the word resilience and self-esteem. I know a lot of people like me who are big and have a very low self-esteem. Whether it be workshops on how to improve your self-esteem that would help.

I- I agree with you, because if we have confidence in our own abilities, it gives us that grit we need to stick to things when times are tough. And dieting is tough. You mentioned that you found MOS staff useful during the program. Did you find yourself leaning on anyone else like family and friends for support?

R- No, not really. Well, my mum has been very good. She has been encouraging. I live a distance away from her and living by myself there isn't anyone really to rely on apart from yourself. So, no not really.

I- Did you think the way that the staff treated you at MOS helped or hindered your progress?

R- It helped, I was going and I was going to the group sessions. I don't know if some more one on one time would have helped as well, because you could share with the group but you sometimes don't know those people. It gets a bit difficult and each time I went there was different people there. Trusting in others and knowing how much you can say and what you feel comfortable with saying was difficult. Maybe a bit one on one, even if it's just a 10min check in to see how you are. You know I got weighed-in and then I went into the groups and no one really knew what was going on.

I- Out of curiosity are you getting one on one sessions now?

R- Yes, I see the therapist once a month and next month I get to see the psychologist. So yes, I am getting that now. It's helping. You see I was struggling with the carbs again. We talked about how we could overcome that, and having oats in the morning has been helpful. It's been helpful talking and getting hints on what to do when I am really tired, I have chronic fatigue as well. When I'm not well I need food, I can just pull out of the freezer, in the past I used to order takeout. So, the idea of having food at home and buying the right groceries so you have food at home, so you can have something low calorie that's quick rather than getting home delivery.

I- So, it seems like you have a few extra tips you've picked up during individual sessions?

R- Yes, it's been really good actually.

I- Did you have any other feedback?

R- No, just more one on one during the initial time would really help. There's only so much you can share in a group and when you are struggling to stay on it, when you aren't doing so well. You know I'd just say, I've had a bad week. Actually, talking about what actually went wrong helped on those few occasions, it was very helpful. It was good to have the group to see what other people were doing, but also having that individual time would have been useful too.

-Interview end-

Date: 2.7.2018

Phone interview ID: 13

Duration: 25min

Participant transcripts

I- How have you been?

R- Just struggling to keep the weight off. I've put some back on, it's frustrating that's for sure.

I- That does sound frustrating. Can I start by asking you to think back, when you were initially told that this service included the use of meal replacements, how did you feel about starting a meal replacement program?

R- I felt ok with it, I had no problem with it at all. I was quite happy to try anything to lose the weight.

I- What were you prescribed when you started the diet?

R- I cut back on half my foods. Cut back on salts, oils and all the food I used to eat. I was doing 3 shakes per day with vegetables.

I- How were your hunger levels?

R- Surprisingly, I wasn't hungry at all. With eating the vegetables in-between, I wasn't hungry at all.

I- How did you find the diet to stick to? Easy or challenging?

R- At first it was a challenge, because I used to see what the kids used to eat and then what I was eating. But then it got easy.

I- What eventually made it easier?

R- What made it easy was seeing the weight come off. It made it easier because it showed that it was working.

I- So are you saying your motivation changed because of the weight loss?

Yeah

I- Did you have any bad days, days when it was hard to follow?

R- Yes, when we had parties. I wanted something extra but knew I shouldn't or with alcohol. But as I said I managed to keep going with it.

I- So when you did have a bit extra at parties, did that little extra continue for days after?

R- No, just at the party. I'd have a little bit too much. But then the next day I would be right back to what I was doing before. I didn't let it go.

I- How much weight did you lose in total?

Participant transcripts

R- In total I lost 41kg in 5-6months. I was doing the 3 shakes the entire time with me vegetables and whatever else. It was just working so well I didn't mind just to keep going with it.

I- **What affected your ability to stick to the plan after that?**

R- My legs, the skin got a scratch on it and it wouldn't stop bleeding, like water. I was losing all the skin on my legs. I had an ulcer. I had to get some meat or iron back into me to help the healing, because that seemed to be the only thing that worked. So we went down to 2 shakes and a meat in between. Then, me and my daughter and I had a car accident in January and I tore my shoulder, the right ligaments. The pain killers and that just put me back on to food. The shakes weren't working with the tablets, I started getting dizzy spells.

I- I didn't know all this had happened…

R- Yeah well, my daughter and I were driving back home from a hunting trip and we rolled the 4-wheel drive. I was grabbing hold of her with my right arm to keep her in the chair and I tore the ligaments in my shoulder. It was good because she only came out of it with a few bruises, and I only had the torn shoulder. The car was a mess though. The police were very surprised that we walked out of it.

I- That would have been so scary…

R- Yes, it was, and the shock of that and the tablets to take the pain away just ruined it for me. But I am back on the shakes now, but it doesn't seem to be working. It doesn't seem to be taking the weight away. I am feeling hungry still, but maybe that's from the pain tablets. I'm not sure.

I- I'm really sorry to hear that.

R- So it was really medical stuff that derailed me in the end. Well, the pain medications for the shoulder don't do anything for me.

I- So, before all this happened, did the way you feel about yourself ever affect your ability to stick to plan? For example, self -esteem, confidence etc

R- No, not really. Before the accident I was happy to do it and to keep on going. I wouldn't have kept on going if it weren't for the pain. I'm only slowly starting to get back on to it now. Hopefully I can start losing it again. I'm a bit frustrated because I've put a lot of it back on.

I- How is your mobility like after the accident?

I went back to work straight after the car accident. I was off for 2 weeks and went straight back to work. I couldn't stand just sitting around. I was there just playing on the lap top.

I- So, it's not lack of movement then that's keeping the weight on…

R- No, well I am back up to doing 6-7000 steps per day, which is what I am recording. That's just what is recording, I probably do much more than that. Maybe 10-11,000. I noticed from the tablets it's making me eat slightly differently to what I want with the shakes.

Participant transcripts

I- What skills do you think are important to be able to stick to the diet plan?

R- Knowing how to cook was one. See, I know how to cook stir fries. That was good to cook my veggies, and I suppose the will to lose the weight because I didn't want to end up like my mum.

I- Were there any other motivating factors that you had?

R- Well, I have my son's wedding coming up next month. I tried to lose weight to look good in a suit for the wedding. I'm back into slowly to lose a couple more kilos before the wedding. That will make me feel a bit better.

I- How did you find the support from the staff at the clinic? Did you find it useful to sticking to plan?

R- The advice from the staff was good for me. It helped me see things to do a bit differently to what I was normally eating. It helped me. I got information on what was affecting and what wasn't. It was eye opening on what I could do and that was good for me.

I- How about family members and friends, did you get support anywhere else?

R- Yes, as soon as they saw the weight coming off me they were quite happy for me to continue doing it. There was a lot of support there for me.

I- What did they do for you exactly?

R- Just encouragement, no one helped me with cooking or anything like that. I know what they are like with their salts and oils.

I- In terms of cost, did you find it hard to purchase products?

R- When compared to what I normally spent on food, it was cheaper. Where Jenny Craig cost me a lot more than what it did with you guys.

I- And how about compared to the normal way you used to eat?

R- Well, that even came down for me. As I said with Jenny Craig it was a lot more expensive than doing your program. See, even on your program, I was still within the family budget as far as buying food for my kids as well.

I- How about the groups?

R- They were good. I learnt a lot from hearing from other people about how they were managing their diet and struggles that others had too, which is the same struggles I had. I guess that helped me too. It helped me to keep going.

I- Did you end up doing any individual sessions?

R- No, I came to that course where you had a bigger group. But that's when I started working away from home, like in XXXX and XXXX.

I- How did you go with travel when you were doing the program? Did you travel and work away from home then as well?

R- No, I was in XXXX. I had problems with my legs then. So they wouldn't let me travel. I think the traveling away from home might affect me this time around sticking on the diet. Just the anxiety of leaving the kids back at home by themselves while I am away. That might be an issue next, being away from home.

I- Worrying about the kids, do you do a bit of emotional eating because of this?

R- No, not so much eating. But I do have a drink or two.

I- Any other feedback you'd like to share?

R- No, I was really happy with the program. I really quite enjoyed it. It was good to get the motivation to keep going. I was quite happy.

---Interview end---

Date: 9.7.18

Phone call ID: 14

Length of interview: 20min

I- When you were initially told that this service included the use of meal replacements, how did you feel about starting a meal replacement program?

R- I was a bit unsure at first, but then I was ok because I had done it before. I was worried though that it wouldn't work. Because I had tried different shakes before and they hadn't worked.

I- What do you mean by they hadn't worked? Do you mean that you didn't lose weight?

R- Well, yeah. I tried Isagenics and Tony Ferguson. There's heaps I can't remember the name.

I- Did you try them on your own or did you have help?

Participant transcripts

R- With Tony Ferguson I got help from the dietitian, but with the others I did it on my own.

I- Comparing with how you did it in the past and what you did with us, what do you think was different?

R- I think because doing the groups and getting weighed properly and seeing other people who were just like me, made me realise; hey we are all in the same boat. The support there was a lot better, that out there. With the other programs they just sent you along your way. But with this program you got to talk about things. Like what you did good that week and what you could do better.

I- So, you are saying support was a big factor in your success?

R- Yes

I- Did you ever feel like dropping out of the program?

R- Yes, well maybe at the beginning. I hung in there, because I didn't think I could do it. But I got down to a very low weight. I have been challenged with going from a big weight, to a low weight, to getting back to my starting weight. I am now hoping that I have gone back down a bit, but I don't know. I am going to get weighed this week.

I- How much weight have you lost?

R- Maybe close to 18kg. But I put all that weigh back on.

I- How do you think the weight cycling you described affected your ability to stick to the plan?

R- I think that it kept me from returning straight away. But I could see that I just kept putting weigh back on and I didn't like that. My clothes didn't fit, compared to where I was. I liked the feeling when I lost the weight, so I thought I'd get back into it.

I- What skills do you think are important to be able to stick to the diet plan?

R- You need to be motivated to do it. You need to have full concentration, forget about everything else and focus on what is better for your health. Knowing about food helps too.

I- How did knowing about food help?

R- Yes, in the beginning I was getting recipes and at that time it helped. I found out how to eat vegetables differently and at that time I was eating kale and I lost so much weight, just eating the kale.

I- Sounds like you stuck to the plan, did you ever have any bad days where it was difficult to stick to?

R- No, not really. Well, I guess you know you some days where you feel down. People look at you because I'm big, and you feel down.

Participant transcripts

I- Sound like when you felt stigmatised it changed your mood. Did that affect your ability to stick to a weight loss program?

R- Stigma did affected me by feeling really upset, I didn't want to go out. People look at you like you're an alien at the shops, so I socially isolated. That made me start emotional eating again, and that made me put on more weight. Then my Dr put me on to this program. Because of all the negativity and the way people looked at me, the first couple of times I went down to the program I was embarrassed to be there. My family had to push me to go because I didn't want to be there. I was too embarrassed.

I- **I know there was a stint where we didn't see you much. What happened during that time?**

R- Recently we had a personal experience where we became homeless, that made me fall off track and I wasn't able to concentrate my mind was elsewhere. I forgot all about the program and I had nowhere to cook. We were living in a motel and just living off what we could. It was very hard money wise.

I- When you say, it was hard money wise, do you mean hard in general or hard to purchase the products?

R- Well, both I think. The meal replacements aren't cheap and we really couldn't afford much at the time.

I- Was there anything else you'd like to add?

I thought that doing the groups was really helpful and seeing you guys regularly helped. I was coming fortnightly and turned it up to weekly. I prefer the fortnightly visits.

---Interview end---

Date: 16/7/18

Phone interview ID:15

Duration: 29min

I- When you were initially told that this service included the use of meal replacements, how did you feel about starting a meal replacement program?

R- Negative.

I- Any reason why?

Participant transcripts

R- Well, you only have a certain amount of fibre in your body. After a few days your outputs are different, you have a bit of a hollow stomach after a couple of days.

I- Had done the meal replacements before?

R- Yes, about 15 years ago at the Metabolic Clinic. I did it under professor XXXX.

I- When you did start the meal replacements in the groups, what did you end up doing?

R- I don't think we even started it. I think we just tried to modify the sugars in what I ate from what I remember. This is going back now. So it's hard to remember. We are trying to work around the problems with the chronic seizures. I never really felt satisfied on the shakes. But then I am not a chronic eater. There are times I get tired and lazy due to seizures and I am always looking for a pick me up.

I have a milk allergy. So if I have milk for a few days in a row I start to cough. If I only have it for 2-3 days I'm ok. The meal replacements must have milk in it, because I could feel it.

If I could get my seizures under control it possibly it would be different.

**Participant describes his current journey through medical tests and extended medical professionals and specialist who have not been able to diagnose him with a disorder nor find an issue. They have suggested psychological therapy and the patient does not agree this is the type of therapy he needs.

I- So, during that time did you try having the meal replacements at all?

R- Yes, I actually went and got a rice based one, that didn't have any milk in it. What I was doing with XXXX, in the initial stages with XXXX, now I remember. I was having one of those protein meal replacements, the brand XXXX suggested. I remember chasing it up and I tried it and got caught. I started coughing again. So then, I was just having one every 2-3 days and I was ok.

I- Did you see any results from doing that?

R- I can't remember what the scales where doing, but I am sure it's reflected in the notes you have. I think it came down only 1-2kg.

I- At any time during the program, did the way you feel about yourself impact on your ability to stick to the meal replacement plan?

R- Yes, I do. Because I was in a bad time with the seizures when I came to see XXXX. All that balances it out and it affects you one way or another. My blood pressure fluctuations prevent me from being able to stick to any diet anyway. I know my doctors says it has nothing to do with it, but I know it does. I can feel it. I've stopped trusting in so called experts, you know yourself better than any doctor could anyway.

Participant transcripts

I- Thinking about the staff at MOS, do you think you get the right support from the staff at the clinic for help with diet and weight loss?

R- Yes, I think the information is right, but I disagree with the concept that you have to white knuckle it, and I hear that a lot. I understand that some people are quite soft on themselves. But if you look at my psychological profile I tend to be hard on myself and I just know something is going on. You see I was eating these biscuits after feeling nauseous and having craving for them every night, and when I went to see Dr XXXX I mentioned that to him and the junior doctor that was with him. I wanted to see if I could get rid of the biscuits and have at least a 16hour window of not eating. He said that's GORD. I thought it could be, but I never felt it as a burn. It just felt like the nerve was alive running straight down my body.

R- What happened after that was I ran out of Panamax. It was about 2-3 days that I ran out and I never really thought much about it. I got back on the tablets when I got some at the shops. Then the pain started again, I never really took notice that it was gone. So, it was the Panamax that was giving me this burn, that I never knew was GORD. I felt like a nerve pain running through my body. So, I stopped taking it, and bam, no more biscuits at night. Yet, white knuckling it, was the answer given to me to get off the biscuits. But now I know the biscuits was due to this pain.

R- That's what the Dr said too. He said people have biscuits sometimes to get rid of the pain. After I told so many people this, he was the first person to give me the answer. I don't think its GORD, but you have given me something to think about. It was only just by accident that I ran out of Panamax that night. So now I try to have my meals a bit earlier and stopped taking the Panamax, now I am going without the pain tablets, to get a bigger window between eating.

I- Do you feel like you are listened to by staff at the clinic? Or do you feel like we miss things?

R- I think you go into an automatic reply, like you've heard it before and you put me in the category in trying to lose weight, but not making much of an effort. You know it was bought up that the dietitian, saw me over the other side at the café having a coffee and two sweets and I don't feel bad about that at all. It was a good wake up call. But at the same time, I was just out of my area, I hadn't eaten. I had gone hard at it for the past 2-3 weeks and I just seen that, and thought, I wonder what that tastes like, I'm just going to try. I am interested in cooking and I wonder what is in that?

R- Well, it was a good slap on the knuckles and well done. She told me, and I thought it was great. She told me, look you are coming here and then you are going over there and eating that. It was a good wake up call. But I was just being sorry for myself and that sort of thing happens when you are being hard on yourself.

I- What you are describing is accountability and needing to front up to things…

R- Yes, I am cool with that. I reckon that is great. She told me she was just trying to help me, and saw that as an error in my ways. I took it on the chin. But I have been tacking food for such a long time, I just want to have a breakout every now and again. That's probably what it was, you know I wonder what that is and what it tastes like. I've never seen a sweet like that

before. I love cooking, but cooking for yourself is boring, I usually invite people over and you tend not to do that on a diet.

I- So, from the sounds of things, you tried the meal replacement diet, but ultimately it wasn't for you because of the side effects, taste and it didn't work well with your medical conditions. Have I got this correct?

R- That's exactly right. Some things just don't work out, but I am sure they work for everyone else.

---Interview end---

Date: 20.7.18

Phone interview ID: 16

Duration 21mins

I- How long ago did you do the meal replacement shakes?

R- I really can't remember exactly, maybe last year.

I- When you were initially told that this service included the use of meal replacements, how did you feel about starting a meal replacement program?

R- I felt excited because it was a good opportunity. I wanted to lose weight, just knowing there was a group out there that could actually help was exciting.

I- Had you done meal replacements before?

R- No, I had heard about it, but never tried it.

I- When you were doing the meal replacements, what sort of regime did you follow?

R- It was supposed to be 3 shakes per day and vegetables, and you could have extra vegetables as a snack if you were hungry.

I- What type of results did you get?

R- I did actually lose weight. I think it was 8kg all up.

I- Did you ever have bad days where you couldn't follow the diet?

R- Yes, I did, especially at the time I was dealing with different people. Sometimes I would get sick of it. The other problem I had was going places, because it would give me diarrhoea.

Participant transcripts

I- That sounds unpleasant. Did that stop you from doing the diet?

R- Yes and no, it was more the money issue. It was difficult to afford to buy the shakes. I was going out and couldn't do them. I felt frustrated because I couldn't do them. Yeah, but, I did like the program. I did it on the days I could. But just losing weight, people would comment about how you looked and that boosts yourself confidence so made you motivated to do it more. My other issue was going places and not being able to control my bowel at times. So, if was to take it I would be restricted at home in case I needed to rush to the toilet. I also had gout too, so I couldn't rush to the toilet if I needed to. I didn't want to be out and have an accident.

I- Did you suffer from these symptoms before you did the shakes? Or this was a result of doing the program?

R- No, it was from the shakes. It was like a clean out. If I knew I was going somewhere I wouldn't take the shakes that day, but other than that it was fine.

I- Sometimes the shake can have this effect if you have lactose intolerance. Have you ever been told you have lactose intolerance?

R- No, but I don't drink milk because the same thing happens.

I- At the start of the diet, during the groups did anyone ask you about side effects?

R- No, we didn't talk about it. I didn't think it was a big deal anyway. I just thought that's how they worked. I felt like it was helping me lose weight anyway. I just wished I could do the shakes more, because they did help a lot.

I- Ok, well, let me ask you, did that ever impact on your ability to stick to the program?

R- Just the guilty factor when you aren't on the shakes.

I- Did that motivate you to want to do the shakes?

R- Yes, I got motivated because I know I should do them, but then frustrated because I couldn't do them more often.

I- What skills do you think are important to be able to stick to the diet plan?

R- Just being prepared, having meals prepared and planning your day and week ahead. I also think knowing when you are full helps, knowing when and what you can and can't have. Then you know when people offer you food you can say no, because you are full.
Make sure you have the right foods at home. You see that was a big problem for me. Times when you are in a rush, you can't go to takeaway stores because that's all the bad food. I wish they would invent a drive through with healthy foods, that would help! So, taking stuff with you helps.

Participant transcripts

I- You have mentioned a few times family and friends and eating out. So how did you handle social situations?

R- That's the part where I failed because we had a lot of functions on at the time. But I think just being prepared and take your own meals or portion out from the food available what you can have. Just know not to overdo it. But a lot of the time there is the temptation there.

I- Did you ever try taking your own food with you?

R- No, I just picked. So, if they had salad or seafood like prawns I would just have little bits of things. It's all about the portion sizes that matter.

I- So, what you are saying is that because you knew what foods you could and couldn't have, you knew what to pick when you were at parties?

R- Yeah well, being on the program we were given a list of foods we can and can't have. That helped. It taught you what kind of impact you can take from different foods. Yeah, we had a food chart to follow, for example you could have a lot of salad, as much as you liked, and a little bit of protein.

I- How did you find the support from staff at the clinic, in regards to assisting you stick to the plan?

R- I came into the clinic with a few issues and being in the clinic actually helped me. You see I had sleep apnoea and problems with periods, and just having the doctors get me to go see different specialists, like my sleep apnoea I have it under control now. So I sleep with a mask, and I had a Marina surgery done and the bleeding stopped. I was bleeding constantly so it was hard for me to do exercise, I just wasn't confident enough to go out. Like going to exercise at the swimming pool and that was not even an option. But after getting the Marina done, from Dr XXXX referral my bleeding stopped so I can function a little bit better. I'm exercising. And just with the sleep apnoea as well, I am now sleeping better. So just being able to see the right people to help me with weight and health issues was useful. So now that that's all fixed I just need to concentrate on my weight.

I- How much weight did you keep off?

R- I am 193kg now. When I first started the clinic, I was 229kg.

I- You have done brilliantly well then?

R- Yes, and it has boosted my confidence and now I am working. Its only part time, but it's got me out there and socialising and walking.

I- Comparing your life prior to doing the diet and now, how do you think your life has changed?

R- My life is so much better. As I said I am socialising and working, and I am hoping it will just continue like this.

Participant transcripts

I- Anything else you'd like to add?

No, everything is fine. I just wanted to say that I always feel good coming into clinic and also when you leave because the staff are so friendly. And that's good because when you do have bad days, you know I came in one time and didn't lose anything. And XXXX said, look it's fine at least you're not gaining. It's a good motivation. At times you sit there and think, I'm not going to go because I've had a bad week, but then like XXXX said we aren't your parents or your teachers, we are just here to support. I know a lot of people don't come to appointment because they have had a bad week, but I think you know, just go. Because at least then you guys can help us. If that way doesn't work, we will just try another way. That's why I like coming.

---Interview end---

Date: 27.8.18

Phone interview ID: 17

Duration: 36

I- Are you doing anything for weight management at the moment?

R- I am doing a mixture of things at the moment. I'll go through what I'm doing. I have a shake in the morning Optifast or I can't remember the name. Then I have a Proslim bar during the day. I have 4 bars generally, and I have vegetables cooked up. I have 3 meals of vegetables, and I haven't gone into ketosis. I might have some protein with the veggies as well, like chicken or seafood.

I- How long have you been doing this?

R- Since 30th Dec I had 2.5 months, were I only did the shakes and bars, and 2 serves of veggies only. Then after 12 weeks I started adding a little bit of protein to it. But I didn't go into ketosis at any stage.

I- What makes you say that?

R- I got tested, and I am hungry. It's been a steady decline in weight, like watching grass grow kind of way. But I am happy with it. There wasn't any dramatic loss of weight. I came in and got tested by one of the people in the clinic. I got a blood test and a breath test.

I- It's interesting, because the researchers found that people weren't going to a severe ketosis it was a mild ketosis during the program. Even though they were still losing weight.

Participant transcripts

R- Yes, well I was really ridged in the first 10-12 weeks. The diet was working for me because I was losing weight, albeit slowly. It wasn't in the way that I had read about, you know how you're supposed to lose heaps of weight when you go into ketosis. XXXX thought, that maybe because I had a gastric lap band for a number of years maybe that had affected something and impacted on the way my metabolism worked. But there may be many explanations I suppose. But I thought, instead of being disappointed because the weight is coming off slowly, think of it in a positive way. The weight is still coming off and it's probably better to come of slowly.

I- When did you have the band taken out?

R- It was about 3 years ago. I had one a long time ago, probably 10years ago and I had problems with a hiatus hernia and had my gall bladder removed. So, they decided to replace the band as well, replace it with a new type of band. But then I was sick in the guts for ages, but no one could explain why I was sick. They think it may have been because of adhesions caused by the band. But it didn't quite explain my gut issues, when the band was removed they kind of improved over time anyway. I was happy enough without it, it wasn't doing much anyway and I didn't want to tighten it because of all the problems I had been having.

I- How did you find the two experiences between the band and the meal replacements in terms of hunger management? Now that you have experienced both?

R- With the band when I had originally had it, it was very successful, I lost 50kg. I wasn't hungry and I didn't crave anything, so that was good. But then that tapered off and I found I could cheat with it a bit. Then I got the new band it wasn't quite as severe as the first one. It didn't take away my desire to eat. But with this I am actually controlling what I eat. You see I just had a month of celebrating my XXXX birthday and retirement and I haven't been as ridged with it. I have been trying to make good choices like order things that are grilled. I have set rules for myself, for example I can't have anything with carbs in it. I can't have anything with sugars or fats in it, but I can have bars. So, I haven't had anything sweet because I know I can just have a bar and I don't crave it either.

R- When I have other food, it doesn't make me think, ah I want to keep having this or anything. So that's good. Basically, it's been fairly easy to go back to what I was doing originally.

I- Did you have any preconceived ideas about starting a meal replacement program?

R- No, I just thought I'd give it a try and see how it goes. I did previously go to the clinic once and that didn't work for me. I didn't find it was a positive experience. Now I am just seeing XXXX once a month. I mean that sitting around discussing every one's problems and issues with food just isn't my thing. I found it depressing. I sure everyone has different experiences and maybe if it was a different group of people, it may have been different. But that didn't work for me, so I contacted XXXX and XXXX about changing to individual appointments. I used to see XXXX way before she had children. I saw her for a very long time, then I had a long gap. But it was useful going back to see her again. She has a lot of history on me, but I know that doesn't work for everyone. But the group support thing just didn't work for me.

Participant transcripts

I- So you did the one initial session in the group, then went into individual sessions?

R- I came out of the group sessions feeling really depressed, and I thought this is really awful. I was with a group of people who weren't in different situation to me, so I just couldn't relate. Maybe with a different group of people, anyway, it didn't work. But it works out for me this way, just dropping in once a month and going over what worked for me and what didn't. My only weakness is cashews, that's my cheat, but I try to keep it under control.

I- Did you have any difficult days where it was hard to follow?

R- On other days, the other day I had a really bad day at work where I normally would have reached for chocolate, I had one of the bars. At least if I eat the bar, I still sticking to the diet. Even if I have 2 or 3 of them in a day. I was able to control it that way, the bars really work for me. That's why I chose them over the shakes. I don't find the shakes very filling. I have one for breakfast of a morning, but they aren't very filling. But the bars allow me to deal with stressful points in my life where I would otherwise reach for chocolate, that I shouldn't have.

I- Sounds like stress and emotions are things that derail you. Are there other things that affect your ability to stick to the diet?

R- My social life might derail me. Trying to maintain my social life and social connection, which often involves food. In the first two and a half months, I had to block myself out. People would ask me if I want to come out, and I'd say why don't we go see a movie instead. I've become better at it, but the diet doesn't allow you to eat out like that. I've become better though at compensating my food choices when out, so now that I can have a bit of protein I have some grilled fish or something like that. I try to choose a plate that has food that I can eat from a Thai restaurant, the choices are limited, but there is stuff I can choose from. So I'd say it's trying to maintain my social life. I do now have 1 glass of wine when I am out socialising with people and try manage it that way.

I- So it sounds like having a bit of nutrition knowledge around food choices has allowed you to circumvent some of the situations that would otherwise derail you. For example, being able to choose appropriate food choices from a menu.

R- Yes, say everyone has had dessert, I wouldn't. I'd hop into the car to go home and eat a bar. That way I wouldn't feel that I'm missing out. I like the Proslim bars, but I don't like any other bar. So, if they didn't have the Proslim bars, I don't know how I would go on the diet. The other bars aren't nearly as nice. I also try to do things like if I am going out, I try to never go out hungry. I'd carry around carrots and things like that. So I try to be very prepared. I also try to cook and have things in the fridge. I'm single, I don't think it would be that easy if I had a family to feed.

R- So I cook up big pots of veggies and things like that and I have them in the fridge. So, for the times I might derail I actually have things in the fridge. I also have things in the freezer for the times I run out of things in the fridge. That was a practice I got into after the gastric

band, having things in the freezer and having things for on- the -go, then you're less likely to get out of control.

I- It sounds like planning was a big factor for you?

R- Yes, and in the beginning, it felt like I was always cooking. You know how when you just cook veggies they go quicker than if you mix them with a pasta or a protein. When you do mixed meals, they seem to last a lot longer. But I seem to just go through them all the time. But now I am doing things like cauliflower rice and those kind of things as well. I have a few dishes that I have been kind of doing. I've got myself into a routine of cooking easy things. I've just been on a weekend away, and everyone knew I was on a diet, I said to everyone what if I made cauliflower soup? And everyone loved it anyway. So that was good. Because I thought don't eat anymore big meals, and we cooked a lot of meals at home, which was good, but it wasn't perfect. But it was better than a lot of other things we could have been eating.

R- Really in a month of celebrating my birthday, having lunches and farewells from work, and a weekend away, plus drinking gin and tonic. I only gained 2kg and I lost in on the first week back on track. I was really happy that I could kind of have those one offs and still be ok.

I- You've mentioned your social life a few times now, so do you think people around you have been more of a help or hinderance with sticking to the program?

R- They are very supportive, extremely supportive. They are happy to go where I suggest. I ask them can I choose where we eat? And they are happy to go with it. I just tell them I can have something grilled.

I- What type of support do you find helpful? Is it the encouragement or simply being open to allowing you to choose the restaurant you eat at?

R- I am not big on encouragement. When I had my lap band, I never really told anyone. Well, after a while, it was only a few select people because I didn't want to talk about it and get into lengthy conversations about it. Even now people say, 'oh you've lost weight', it's nice that they notice. But I don't really want to have a conversation about it. Whereas just getting support by being flexible in what I am doing, is the best encouragement for me. But other people around me do want to discuss things, but I figure it's just my business that no one needs to know about. That's part of the reason why I didn't like the groups. I feel like it's my business. I didn't want to discuss my weight issues in an open forum, and the same with my friends. I don't want to have a discussion about it. But I have told them this time that I'm on this plan.

I- Did the way you feel about yourself (good or bad) impact on your ability to stick to the meal replacement plan?

R- I've always had a pretty high self-esteem. I actually feel fitter and lighter and clothes fit better. And I knew, because I had a knee replacement, but I don't have anything like diabetes or heart problems or blood pressure, but I do have physical issues, like the knee replacement

and precancerous cells in my uterus. There's an element of weight that impacts on hormones, so I knew I had to lose the weight. So I kind of feel like I'm doing something positive. For me it has to be the right time, and I have to make the decision that yes, I am going to do it now and commit to it. I didn't ever feel bad before, but things were harder. I know that if I lose another 20kg things will be even easier again.

I- Did you have a hard time doing exercise?

R- My intention is to do more. I was planning to go to aqua aerobics, my mobility is a bit limited for walking. Now that I am retired I have more time to do some exercise, so it should increase the effectiveness of what I am doing.

I- You said that physical limitations were something that motivated you, rather than any emotional problem?

R- Well, the physical things was why I engaged in the program. That was my motivation. I knew that not being mobile was going to cause problems and moving into retirement not being about to walk will prevent me from travelling. So, I knew I had to get things under control or things were going to be pretty miserable really.

R- There were a few health issues that were causing me to think, that I do have to do something about this now. It's quite nice that I have lost 20kg at the beginning of my retirement, and hopefully by the end of the year that I can lose another 20kg and that will be great. But I didn't have any problems like low self-esteem, especially about my weight. It never got me depressed or anything. That's not why I did it.

I- What do you think about the cost of the VLED program? How did this compare to your weekly grocery bill?

R- It probably the equivalent. It's not cheap buying the bars. I don't know how the people on pensions could afford it. I know that some of the people I came across weren't working, so that might be a bit pricey for them. Its more about the bars and the shakes, when I go to chemist I can easily spend about $180 or something like that with buying the protein and everything. But it's no more than what I would spend normally, but I've always had a good wage and I do have a good super to live off. I've got no concerns. But I think other people may. Also, because I'm single it makes things so much easier. I don't have to consider other people. So I think, I'm in the ideal situation to lose weight. I suppose, other people have families, so they don't need that social contact. Whereas because I'm alone, I can get socially isolated if I don't go out, which a lot of going out revolves around food.

I- Well it sounds like you have come up with a good solution. That you pick certain places and dishes that you can eat.

R- Yes, and I am also trying to space out eating occasions. So, I try not to do so many eating things on top of one another and space them out a bit. It might be once a week to eat out, not 3 nights in a row. That's also how I am trying to manage it.

I- So, a lot of walking gatherings then?

Participant transcripts

R- Yes, that's true! But I do feel like this is something I can do. I must say I do like veggies, a lot more than what I did. So, I kind of feel like I can happily continue with this long term. And I've had discussions with XXXX about any potential effects of not having carbs and eating the bars, and maybe drop bar on how many bars you have, but what are the long term effects on staying on this type of diet long term. But I am much happier doing this.

R- I went to a, well, my knee Dr sent me to another Dr: well I'd had the gastric band and he wanted me to have the gastric sleeve. And I was really unhappy. You know from the band I had all sorts of things happen, and now I have diverticulitis as a result. I'm like, I don't want to be skinny and unhealthy. I knew there had to be another solution. I figured that I had to stop eating anyway, so why not just stop eating without the sleeve? I was quite adamant that I wasn't going to get any other type of gastric surgery. But I am happy this is something that I can do longer term.

R- One thing I do think that is missing from the program, is good recipes. I've looked up the Optifast recipes and they are fairly basic and uninspiring. I do think there is a lack of interesting recipes.

I- Are you talking about vegetable recipes?

R- Yes, vegetable recipes. The Optifast ones were really uninspiring. You kind of have to have enough interest. I am tempted to write a recipe book with all the recipes I've found.

I- Are you tending to follow recipes now or do you just make things up?

R- I tend to make things up, well, I kind of look up things or I take some of the stuff I used to make and modify them in a much more slimming way. I do occasionally do a "chicken curry" and just use the green paste, and put 2 desserts spoons of coconut milk, so it kind of has the flavour but so minimal coconut milk and calories. It would give me a bit of the taste I was looking for. That's what I've been trying to do have things, with a bit more taste to keep it interesting.

I- That's interesting, because many people have been saying they would like recipes, because they couldn't find anything online.

R- Well, that's one thing I would recommend to improve the program, if anything. Is to provide more recipes. Other than that, it was good. I got the help I needed.

---Interview end---

9.2.2020

Participant transcripts

Telephone interview: ID 19
Duration: 35min

I**. When did you start doing the MRP?**

R. I started them over 1 year ago. I did the full 12 weeks, then I went on to a partial.

I. **And from then what have you been doing with your diet?**

R. I've been on and off, but last year mentally I went through a bit of a hard time. I wasn't the worst, but I could have been better.

I. **Did you come off the meal replacements all together?**

R. No, not all together, during the day I would have at least one per day. I'd have one for breakfast, then during the day I wasn't sticking to it at all. I was having the one in the morning and saying to myself "today I'll get back into it", but it just never worked out.

I. When you were doing the intensive and partial meal replacement phase, what made you stick to it?

I had a lot of support from my girlfriend. Support from family with words, but with actions, not so much. I did ask them to maybe keep the biscuits and lollies and stuff in someone's room, but that didn't happen. So, seeing it around all the time in the main cupboard made things difficult. Back then I had a lot of motivation to lose weight. But then things happened, I lost a few family members and got myself in a really bad spot. That's when I started going up and down with my weight. I just needed to talk to someone, and I do sometimes talk with my counsellor at Tafe. So, I went to see her, because I do keep a lot of feelings to myself. I got to the point that I wanted to feel better. I feel like mentally, when I'm in a good spot, you can't stop me. I find that when mentally I'm not great, I look to food to give me happiness. I feel good when I'm eating it but after, I feel like crap. Then I eat again to feel better, and it's a spiral. Now, instead, I go and talk to my counsellor. I go every week, even if I have nothing to say. It's good to just sit and have a chat. When I have a clear head all aspects of life are just easier.

I. How was your girlfriend supportive, what did she do?

R. She gave me tough love just before I started, which I needed. Long story short, she said to me, you need to sort your shit out or I'm out.

I. So, she gave you an ultimatum?

R. Pretty much, and I was like, well this March it would be 10years together. This was when I was the heaviest I have ever been, just before I came here I was 286kg. Then I came here and I was 261kg. I did a little bit by myself, and that was it pretty much. Throughout the whole thing she was always encouraging. If we went out for food it was always to the healthier places like vegetarian or something.

I. So how you said previously your family was encouraging with words but not actions, but she seemed to be both?

R. Yes, which was nice, so we could go out to eat and have a nice night together whilst still sticking to the plan. Which was incredible, it was nice. She's not big, but she's also not the smallest girl, so she was doing it along with me and tried to lose weight with me. It was nice to do it with someone else.

I. You said you had a lot of motivation, what was the motivation?

R. When I was 286kg, after my girlfriend told me that, I didn't realise how unhappy I was and how bad it was. Everything was being impacted, in my personal life- everything. I couldn't do the job I'm doing now the security job, standing for a long time. I could only stand 20minutes at a time, my lower back was in so much pain. It was impossible. I'd have to take breaks. Walking, I'd only last 10min. I couldn't buy clothes, it was shocking. It was so bad, really hard. Before losing weight, I used to just think in my head, "oh I just can't buy clothes, it is was it is" and not think too much about it. I got a bit of a kick up the arse and had a bit of time to think about it. I should be able to go for a walk. I should be able to buy clothes. I should be able to travel see the world. You end up feeling like a bit of a prisoner in your own body. That's when I started looking into talking to people, and started to talk about my feeling to people things started to go better. When I have a clear head, things are just easier.

I. In terms of dropping off the diet, you say depression was a big thing for you. Can you think about anything else that derailed you?

R. It is hard dieting, my family aren't the biggest people, but they do eat really bad. At home in the cupboards and fridge are filled with bad food. Seeing all that stuff in the cupboard on a daily basis makes it very hard. You have to use restraint all the time. See that several times a day, is very, very hard. So, the food environment and depression made it hard to stick to.

My worst thing was depression. It's something I battled with for a while. My worst was 16 to 19 years, just horrible years for me. Horrible years. It's definitely is a lot better now. But 16 to 19 years, it was bad, just every day was bad. Now, it's more like, I have a lot more good times than bad times. I find going to talk to my counsellor once a week, just helps. It really helps. I just try to keep a clear head all the time.

I. What about the service itself, how did you feel about it?

R. It was incredible. I remember my first time coming here, it was nice. It was nice to know, not that I want other people to have trouble with their weight, but it was nice to know that other people had troubles with their weight and just all coming together we had a shared experience. You don't feel alone with it. It's nice to come and be able to talk about things, find out how this person struggled, and that person struggled and how they got through it, it just helps. It's really good coming here.

The group sessions are helpful and I coincided my weigh-ins whenever I went there, so every 2 weeks. Number 1, because the scales I need, need to be at an obesity clinic because I am too heavy. It was good to break it down in those 2-week increments, not to focus on the actual weight coming down, but focus on the strategies to help me eat the right things.

I. How about the way you were treated by staff?

R. Oh, I have nothing bad to say, everyone has been fantastic, they just cared so much. It was so nice. It's a very different group of people that you meet here, people don't judge you.

I. What do you mean by that?

R. When I first started, I didn't want anyone to know I was coming here or doing the diet.

I. Why was that?

R. I think it was shame, but more shame from myself. Coming to a group like this, I think allowing myself to get to such a bad weight point. Now, if anyone asks if I'm doing the shake diet, I tell them – yes. I come to the centre here and I am doing something about my weight. I have no issue telling them everything.

I. What changed?

R. I think it was my mental health. Mentally I am in a lot better place. Some people have asked, like friends ask- they say oh your face looks a little different, and I tell them I've been coming here, dieting and trying to lose weight. Everyone just says, fantastic! Everyone receives the information well. I don't know why I felt that way before. When they do find out, they just say- good for you, you're sorting yourself out.

When I first started, I don't know why I didn't want to know. I guess it was stigma, I don't know. I just didn't want anyone to know. I feel like I just had this thought in my head, like judge me or something. Now it's just like, this is what I am doing, it is what it is.

This program is fantastic, I know I have disappeared at different times. But it is fantastic, it's nice to have someone keep you accountable. Sometimes you come here and you've had a bad two weeks, you come here and you want to do better. You jump on the scales you lose weight, it gives you a boost in confidence. I like coming here.

I- Is there any other feedback you would like to give?

R- No, I think I covered everything

---Interview End---

Date: 24/1/20
Phone interview ID: 20
Interview Duration time: 22min

I. How was your experience at the group program?

Participant transcripts

R. It was good because I did it 2 years before. I got to the groups attended 2 meetings then I fell off.

I. Why did you fall off?

R. I think, I was wasting everyone's time. I wasn't doing it properly. I got overwhelmed, they gave us a hand out to record all of our food, things like that. That time it was overwhelming, but this time it was a bit different I think. It was more just talking about the group, I found it was a bit easier this time. Maybe it was the information I was given or if I was just ready to do it properly. Oh, and I know it's also because last time I was doing the keto diet at the time, because keto was working for me they did encourage me to continue on it but do the shakes. But because keto was working at the time, I didn't do the shakes.

I. Did you lose weight doing the keto diet?

R. Yes, I lost about 20kg doing the keto diet, but once I broke the diet it was really difficult to get back on. But with the shake it's much easier to maintain, it's a simpler diet to maintain. It's been 2.5months for me now on the shakes and once you're in a routine it's much easier to maintain.

I. How much have you lost on the shakes?

R. 44kg so far.

I. Was anything else going on that made doing the diet difficult at the time?

R. I think at the time my home life was a lot better. That's one of the reasons I did seek help. I suffer from depression for quite a while, so both times the reason why I sought help was because I was in a good head space at the time. My depression is linked to my weight, so when my weight deteriorates, so does my depression. When I started to gain weight, that's when my depression came along too. That's when I started to become demotivated to do anything really.

R. That's where I found the drop-in group sessions extremely helpful. I did my weigh-ins whenever I went there, every 2 weeks, so I could see the weight change and also so I could focus on the strategies to help me eat right and talk about the things that made me eat the wrong things. Why my diet was so poor in the first place. So, if I took care of those little things then ultimately, they will help me lose weight. Basically, the weight would take care of itself. Whereas before I used to think, right well this is the weight I have to get down to before I can go back to what I was eating before. Just having the mind set to deal with my problematic mindset that caused me to eat badly in the first place.

I. Tell me more about the depression

What happened to me was, that I was a drug addict when I was a teen, early 20's. I used marijuana to cope, when that stops working, that's when my life starts to spiral and depression manifests itself. Because of my weight I also have sleep apnoea, so when I'm affected by my sleep apnoea my weight is affected and so is my depression.

Participant transcripts

I. Why is that, do you emotionally eat?

R. I'm not too sure if I emotionally eat. I know I've had a bad diet though, I used to eat a lot of meat and bread. I used to not eat during the day because of my pot use, it used to stop me from eating. I used to not eat during the day, then at night I would eat a lot of food. So, everything that I would normally eating during the day I'd eat in one go at night in one sitting, then go to bed.

I- Would you say you, binge ate at night?

R. Yes

I. Putting that aside, now that you say you're doing well on the diet. Have you had any bad days?

R. Yes, I have, and it's been more recently. In the beginning, I was stricter with my diet for at least the first 2 months, but then towards Christmas was probably when I was eating a lot more meat than what I should. In terms of adding carbs, it was only on Christmas day for one meal and recently in the whole 2 months, maybe 3 off meals altogether. It wasn't a lot in comparison to what I used to eat.

I. Why on those days was it harder, was there anything that happened on those days?

R. Probably because I was doing it for so long and the idea it was Christmas, I gave myself the leeway. Nothing emotionally, just wanting it I guess. But in the past when I've done something like that it would actually send me all the way off. Even just eating something in the slightest, I would have just given up. The best analogy I can think of, is if my car had a flat tyre, because it had a flat tyre I'd walk around and start bashing the car. Whereas, this time, the mindset is that, yes, I've had a slip up, yes it wasn't the best and I feel guilty that it happened, but let's get back on.

I. So, you have had a mindset shift?

R. Yes, defiantly. In the past when I have started something like this, it was all or nothing. I think also I've had to use the same mindset with my recovery. I've been clean for over 1 year XXXX, it's the same mind set. Once I broke, I'd start using again, but thankfully I haven't broke with my recovery. I had the same mind set, yes things may not be ideal, but I have to keep moving forward. Get back on the diet and just do my best from then on. Wipe the slate clean and start again.

R- So what I did was when I first started the diet I was on 4 shakes and 60g of protein powder or between 100-300g fish or lean meats. Then I got down to 3 shakes, then eventually I got down to 2 shakes. But that's not what she wanted. I did ask her, but I think she misunderstood what I said, so I got down to 2 shakes per week for a week or two. When I went back to see her and explain to her she said, no, please go back up to 3.

So then at Christmas time when I ate what was less than ideal. I went back to 4 shakes and started back at the beginning again. That helped me get back into the mindset again. Since Christmas I haven't been as strict as I first started.

I. **Reflecting back on past experiences, really helped you navigate through the barriers you had that would have normally derailed your diet in the first place.**

Going back to the topic of the group sessions did you have any problems with other people being there in the group setting, in regards to listening to other people's problems and the depression?

R. Not at all for me. Mainly because I was there for myself, so listening to other people's journey and what was going on in their life didn't really concern me, because I was focused on what was going on in mine. It was interesting to hear their perspective and opinion but I was focused on myself.

I. **Where there any skills you developed attending the groups?**

R. Yes defiantly, the information was key. So being able to check in and ask questions to a dietitian every two weeks has I progressed or as I got better at things, being able to ask someone about it was a huge help whereas if your only source of help is a Facebook group or online there a lot of conflicting information. You have to wade through the BS. I listened to the dietitian, it was easier.

I. **Was there any other things you wanted to add?**

R. For me routine was important it kept me on it. When the routine was mucked up during the Christmas period that's when things got a bit mucked up for me. I am looking forward to the kids going back to school so I can go back to my normal routine.

---Interview end--
