# Supplementary material for: Dietary adherence and program attrition during a severely energy-restricted diet among people with complex class III obesity: A qualitative exploration
Source: PLoS One. 2021 Jun 17;16(6):e0253127. doi: 10.1371/journal.pone.0253127 (PMC8211265; doi:10.1371/journal.pone.0253127)
Supplement: Attachment — Submitted filename: Response to reviewers PLOSONE_G.MASTON R2_V1.docx [file pone.0253127.s004.docx]

**Detailed responses to the comments of the Reviewer**

# Reviewer #1

1. Line 152: please use the full company names i.e. Cambridge Weight Plan Ltd as the descriptor not Cambridge.

**Our response:** Thank you, the manuscript has now been amended accordingly starting from line 291.

2. *Line 176-180: With the greater explanation of the dietary methods this highlights a question regarding your primary outcome which was dietary adherence - Was there a difference in views between the group that had food and those that chose to just have the meal replacements only? Maybe suggest add a little clarity in the discussion.*

**Our response:** Participants were specifically asked about the addition of protein and the mode in which it was taken during the SERD, however, it did not illicit a conversation about food-based protein and adherence. Participants who used food-based protein fell into both groups; those that were adherent and those that were not non-adherent. This has now been added to the discussion section of the manuscript, starting at line 1193.

3. *Line 729: Your comment regarding “The interviews did not reveal internalized stigma as a factor in poor dietary adherence” is questionable. You did not measure internalised weight stigma using a questionnaire such as Weight bias internalisation scale and did not ask specific questions related to this, therefore how do you know? People living with obesity are often unaware of the internalisation of the weight stigma, as is internalisation of negative social stereotypes such as people with obesity are lazy, gluttonous etc. So, it would unlikely come out in the interviews unless specifically addressed. This is more likely to be a limitation of the study and suggest removing this sentence and add to limitations.*

**Our response:** Thank you for this helpful feedback. Line 729 has now been removed and this limitation has been added the discussion section of the manuscript line 1307.
